# Behavioral and neuropsychiatric challenges across the lifespan in individuals with Rubinstein-Taybi syndrome

**DOI:** 10.3389/fgene.2023.1116919

**Published:** 2023-06-21

**Authors:** Dima Qu’d, Lauren M. Schmitt, Amber Leston, Jacqueline R. Harris, Anne Slavotinek, Ilka Riddle, Diana S. Brightman, Brittany N. Simpson

**Affiliations:** ^1^Division of Human Genetics, Cincinnati Children’s Hospital Medical Center, Cincinnati, OH, United States; ^2^Division of Behavioral Medicine and Clinical Psychology, Cincinnati Children’s Hospital Medical Center, Cincinnati, OH, United States; ^3^Department of Pediatrics, University of Cincinnati College of Medicine, Cincinnati, OH, United States; ^4^ University of Cincinnati College of Medicine, Cincinnati, OH, United States; ^5^Department of Neurology, Johns Hopkins University School of Medicine, Baltimore, MD, United States; ^6^Division of Neurogenetics, Hugo W. Moser Research Institute, Kennedy Krieger Institute, Baltimore, MD, United States; ^7^Division of Developmental and Behavioral Pediatrics, Cincinnati Children’s Hospital Medical Center, Cincinnati, OH, United States

**Keywords:** Rubinstein-Taybi syndrome, behavior, anxiety, age-related change, adaptive living skills, obsessive compulsive disorder, epigenetic

## Abstract

**Introduction:** Rubinstein-Taybi syndrome (RSTS) is a rare congenital disorder characterized by developmental and intellectual disability, broadening of thumbs and halluces, and characteristic facial features. Pathogenic variants in *CREBBP* lead to RSTS type 1 (RSTS1) and in *EP300* lead to RSTS type 2 (RSTS2). Individuals with RSTS can demonstrate a variety of behavioral and neuropsychiatric challenges, including anxiety, hyperactivity/inattention, self-injury, repetitive behaviors, and aggression. Behavioral challenges are consistently reported as one of the primary factors impacting quality of life. Despite the high prevalence and morbidity of behavioral and neuropsychiatric features of RSTS, a paucity of data exists regarding its natural history.

**Methods:** To better understand the neurocognitive and behavioral challenges faced by individuals with RSTS, 71 caregivers of individuals with RSTS, ranging in age from one to 61 years, completed four questionnaires measuring obsessive compulsive disorder (OCD)-like symptoms, anxiety, challenging behaviors, and adaptive behavior and living skills.

**Results:** Results revealed a high prevalence of neuropsychiatric and behavioral challenges across ages. We found specific challenging behaviors were worse in school age individuals. Scaled adaptive behavior and living skill scores differed across ages with an increased gap between typically developing peers becoming more apparent at older ages. Between types, individuals with RSTS2 had better adaptive behavior and living skills and less stereotypic behaviors but higher social phobia than individuals with RSTS1. Further, female individuals with RSTS1 appear to have increased hyperactivity. However, both groups had impairments in adaptive functioning compared to typically developing peers.

**Discussion:** Our findings support and expand previous reports of a high prevalence of neuropsychiatric and behavioral challenges in individuals with RSTS. However, we are the first to report differences between types of RSTS. Further, age-related differences were seen with higher challenging behaviors within school-age individuals, which may improve over time, and lower adaptive behavioral skills compared to normative scales. Anticipation of these potential differential challenges across age is vital for proactive management for individuals with RSTS. Our study underscores the importance of enacting neuropsychiatric and behavioral screening earlier in childhood so appropriate management can be implemented. However, further longitudinal studies in larger cohorts are needed to understand better how behavioral and neuropsychiatric characteristics of RSTS evolve over the lifespan and differentially affect subpopulation groups.

## 1 Introduction

Rubinstein-Taybi syndrome (RSTS) is a rare genetic disorder with characteristic clinical features, among them a distinctive craniofacial appearance, global developmental delay and intellectual disability, and broad and angulated thumbs and halluces (Rubinstein & Taybi, 1963; Al-Qattan et al., 2019; Stevens, 2019). The molecular mechanism was first described in the 1990s when patients with deletions of 16p13 were reported with subsequent discovery of pathogenic variants in *CREBBP* (Imaizumi & Kuroki, 1991; Lacombe et al., 1992; Tommerup et al., 1992; Breuning et al., 1993; Masuno et al., 1994; Petrif et al., 1995; Spena et al., 2015). RSTS type 1 (RSTS1) is caused by deletions or pathogenic variants in *CREBBP* and accounts for approximately 50%–60% of individuals with RSTS (Petrif et al., 1995; Bartsch et al., 2005; Fergelot et al., 2016; Stevens, 2019). RSTS type 2 (RSTS2) is caused by pathogenic variants in *EP300* and accounts for approximately 8%–10% of individuals with RSTS (Roelfsema et al., 2005; Negri et al., 2015; Fergelot et al., 2016; Stevens, 2019). Approximately 30% of individuals receive a clinical diagnosis of RSTS based on phenotypic presentation with no identifiable pathogenic variant in *CREBBP* or *EP300* found (Bartsch et al., 2005; Stevens, 2019). *CREBBP* and *EP300* encode histone acetyltransferases, essential for typical human development through epigenetic regulation (Ogryzko et al., 1996). Given the important role of these genes within signaling pathways, it is no surprise that heterozygous pathogenic variants in *CREBBP* and *EP300* cause atypical development and pleiotropic clinical effects. Although RSTS1 and RSTS2 result in similar phenotypic features, individuals with RSTS2 show distinctive differences, including decreased incidence of thumb and hallux angulation and reduced severity of developmental delay (Fergelot et al., 2016; Cohen et al., 2020).

Children with RSTS demonstrate a variety of behavioral difficulties, which are known to impact quality of life (Stevens, 2019). Early studies have shown that children with RSTS have a friendly and excitable personality accompanied by hyperactivity, significant emotional dysregulation, short attention span, self-stimulating behavior, and difficulties in planning and executing motor acts (Rubinstein & Taybi, 1963; Gotts & Liemohn, 1977). Behavioral challenges are reported consistently but at varying rates (Boer et al., 1999; Galéra et al., 2009; Stevens et al., 2011; Douzgou et al., 2022). Difficulties in attention, social skills, and global functioning as assessed by the parent-report measure, Child Behavior Checklist (CBCL), have been previously reported in children with RSTS ages 1.5–18 years old (Achenbach & Ruffle, 2000; Ajmone et al., 2018). Differences between males and females with RSTS have not been reported within studies utilizing validated developmental and behavioral measures; however, one past study showed some behavioral aspects were more common in males including problems shopping, creating chaos, mood abnormalities and deliberately destroying things (Boer et al., 1999). Further, within our center’s support group, our team has anecdotally seen a greater number of family questions and support needs within the adult female individuals with RSTS. Despite this possible difference and our anecdotal observations, to the best of our knowledge, no systematic study to date has compared RSTS neuropsychiatric and behavioral characteristics by sex. Further, no study has adequately assessed if there are differences within neuropsychiatric and behavioral challenges between types.

More recent studies in children with RSTS corroborate known behavioral findings and add another prominent psychological phenotype – anxiety (Verhoeven et al., 2010; Waite et al., 2015; Crawford et al., 2017; Stevens, 2019). Specifically, Crawford et al. (2017) found children with RSTS have similar levels of anxiety on the panic attack and agoraphobia and obsessive compulsive subscales of the Spence Children’s Anxiety Scale – Parent Version compared to children diagnosed with anxiety disorder. Likewise, Obsessive Compulsive Disorder (OCD), characterized by recurrent and disturbing obsessions and repetitive behaviors, is often a concurrent diagnosis with RSTS and can interfere with an individual’s ability to interact with others and complete daily living tasks (Stevens et al., 2011; Goodman et al., 2014; Wiley, 2020).

Although challenging behavior and anxiety seem to be key clinical phenotypes in RSTS, less is known about how these clinical features look across the lifespan and into adulthood. Yagihashi et al. (2012) surveyed 63 individuals with RSTS using the CBCL and found that challenging behaviors seem to emerge when individuals with RSTS begin puberty. Specifically, they found that the older group (≥14 years) had higher scores on the anxiety/depression and aggressive behavior subscales of the CBCL than the younger group (≤13 years). A recent study conducted by Giani et al. (2022) also used the CBCL and found that anxiety is more prevalent in older children with RSTS in a survey of groups from infancy (1–2 years) to adolescence (12–17 years). Furthermore, prior reports indicate adults with RSTS may experience mood disturbances and anxious, repetitive, obsessive compulsive-like, and self-injurious behaviors (Levitas & Reid, 1998; Wiley et al., 2003; Stevens et al., 2011; Douzgou et al., 2022). Consequently, these findings suggest behaviors reported in adults with RSTS differ compared to the sociable, friendly behavior historically reported, though in cohorts of younger children with RSTS (Boer et al., 1999; Galéra et al., 2009; Moss et al., 2016).

Ultimately, no clear conclusions can be drawn regarding the full natural history of behavior seen in individuals with RSTS based on existing literature. Without this vital information, proactive management and anticipatory guidance measures cannot be enacted adequately to prepare parents, caregivers, or affected individuals for possible changes to minimize impact to social ability and completion of activities of daily living for optimal outcomes (Stevens et al., 2011; Goodman et al., 2014; Wiley, 2020). Thus, the overall aim of our study was to better understand the neuropsychiatric and behavioral phenotype of RSTS, including any age-related differences, from childhood through adulthood, between males and females, and between RSTS types to inform intervention and treatment practices for both families and providers.

## 2 Materials and methods

### 2.1 Participants

Participants were recruited through the Epigenetic Syndromes Clinic at Cincinnati Children’s Hospital Medical Center, RSTS Facebook support groups, the RTS-OKI Family Support Group within the University of Cincinnati Center for Excellence in Developmental Disabilities, and the Epigenetics Clinic at Kennedy Krieger Institute from May 2021 – August 2022. Eligible participants had either a molecularly confirmed or clinical diagnosis of RSTS reported by caregivers as determined by a geneticist and availability/review of medical records and photos. Genetic testing records to confirm diagnoses were able to be obtained from caregivers and electronic medical records with 33/36 reports (92%) available for those reporting RSTS1 and 19/19 (100%) of individuals reporting RSTS2.

### 2.2 Measures

Standardized parent-report assessments described below as well as demographics, clinical history, and molecular/genetic information were obtained from all caregivers and stored on the Research Electronic Data Capture (REDCap) platform. See Supplementary material S1 for clinical history information. All surveys were administered through REDCap with exception of the Vineland-3 Assessment, which was administered through Pearson’s Q-global online platform. Because RSTS is a rare disorder, standardized assessments were used outside their validated age ranges, consistent with previous studies in RSTS and other similar genetic disorders (Yagihashi et al., 2012; Equit et al., 2013; Crawford et al., 2017; Chromik et al., 2019; Baker et al., 2020) with subsequent analyses excluding individuals younger than the validated cutoffs. Scoring methods for each measure are detailed in Supplementary material S1.

#### 2.2.1 Obsessive compulsive symptoms

To measure the presence and severity of OCD-like symptoms, caregivers completed the Yale-Brown OCD Questionnaire (Y-BOCS), validated in ages 6+ years. The Y-BOCS has a total of 10 questions that yield 2 subscores (obsessive thoughts (5 items) and compulsive behavior (5 items)) with a summed total score. The total score determines the approximate index of overall symptom severity, which is delineated into the following categories: subclinical, mild, moderate, severe, and extreme (Goodman & Price, 1992). The Y-BOCS symptom checklist was excluded from our survey battery due to our desire to screen for overall OCD-like symptoms rather than categorize subtypes of OCD. Further, we wanted to be mindful of our participants’ time to complete surveys.

#### 2.2.2 Anxiety symptoms

To measure the presence and severity of anxiety symptoms, caregivers completed the Spence Children’s Anxiety Scale – Parent Version (SCAS)*,* validated for ages 6–18 years old. Survey respondents were asked to rate how their family member would likely behave in the manner described within the 38 scenarios of the SCAS. The subscales are as follows: panic attack and agoraphobia (9 items), separation anxiety (6 items), physical injury fears (5 items), social phobia (6 items), obsessive compulsive (6 items), and generalized anxiety disorder (GAD)/overanxious disorder (6 items) (Spence, 1999; Nauta et al., 2004).

#### 2.2.3 Challenging behaviors

To measure a broad range of challenging behaviors, caregivers completed the Aberrant Behavioral Checklist - Second Edition (ABC-2), validated for ages 5 to 90+ years old, to measure the presence of 58 specific challenging behaviors. The 58 behaviors assessed are divided into five subscales: irritability (15 items), social withdrawal (16 items), stereotypic behavior (7 items), hyperactivity/noncompliance (16 items), and inappropriate speech (4 items) (Kaat et al., 2014).

#### 2.2.4 Adaptive behavior and living skills

To measure adaptive behavior and living skills, caregivers completed the Vineland-3 Comprehensive Assessment Parent/Caregiver Form (Vineland), validated for ages 0–99 years old. The Vineland yields a composite score called the Adaptive Behavior Composite and quantifies behavior and skills within 3 domains and 9 subdomains (381 items). The domains (subdomains) are as follows: Communication (Receptive, Expressive, and Written), Daily Living Skills (Personal, Domestic, and Community), and Socialization (Interpersonal Relationships, Play and Leisure, and Coping Skills) (Sparrow et al., 2016).

### 2.3 Missing items

To account for missing items on specific measures, we computed prorated scores for the Y-BOCS and ABC-2. The second item of the Y-BOCS and 35th item of the ABC-2 were missing initially from the REDCap survey due to human error, affecting the Y-BOCS obsession subscale and total scores and the ABC-2 stereotypic behavior subscale. Upon discovery of the missing items, our team requested these values from parents/caregivers. Prorated raw scores were calculated using (1) or (2) (Aman & Singh, 2017).

$$Prorated\;raw\;score=\frac{Total\;number\;of\;items\;on\;subscale}{Number\;of\;completed\;items}\times Total\;score\;for\;subscale$$ (1)

$$Prorated\;raw\;score=\frac{Total\;number\;of\;items}{Number\;of\;completed\;items}\times Total\;score$$ (2)

Further, there were other measures with sparse and sporadic missing values, as anticipated with study approach. Upon closure of the study, final items without values were given a score of 0.

### 2.4 Statistical analyses

Statistical analyses were run using SPSS software version 19. Descriptive statistics, including mean, standard deviation, and range of raw values (unless otherwise noted) were generated for each score or subscores of the assessments. Given our primary research question regarding age-related differences in clinical phenotypes, descriptive statistics were provided for the overall sample as well as split into groups based on the participant’s age as follows: 0–5 years (early childhood, *n* = 15), 6–12 years (school age, *n* = 22), 13–21 years (adolescence and early adulthood, *n* = 18), and 22–61 years (adulthood, *n* = 16). To probe age-related effects as previously indicated (Yagihashi et al., 2012), we examined linear and non-linear regression models for each variable and determined which model best fit the data. We compared linear, logarithmic, and quadratic models, and determined the best fit model based on significance, *r* ^2^ value, and visual confirmation. To compare age associations further, we conducted separate Analysis of Variances (ANOVA) tests for measurement scores/sub-scores across age groups as a between subjects factor (Early Childhood vs. School Age vs. Adolescence/Early Adulthood vs. Adulthood). Since this is one of the first studies of its kind in a rare condition such as RSTS, we included between subjects factors in separate ANOVAs rather than interaction models to be the most clinically meaningful. We examined the following between subjects factors: Age Group (Early Childhood vs. School Age vs. Adolescence/Early Adulthood vs. Adulthood), Sex (Female vs. Male), and RSTS type (RSTS1 vs. RSTS2). RSTS type was limited to RSTS1 (Median = 10.70 years) and RSTS2 (Median = 8.90 years) within the type comparisons given the inherent ambiguity of the clinically diagnosed RSTS group with its skewed older ages (Median = 34.74 years). In addition, because only one individual with RSTS2 fell in the 22+ year age group, we limited age group versus type analyses with ANOVAs to early childhood, school-aged, and adolescence/young adulthood. Given our cohort included individuals outside of the validated age ranges for utilized measures, we performed initial analyses excluding individuals younger than the validated age ranges as outlined above to ensure younger individuals were not introducing significant bias to our findings. However, secondary analyses were performed for the entire cohort given our small sample size and the overall paucity of such data within the scientific literature.

Significant age-group effects were probed with *post hoc* t-tests corrected for multiple comparisons. Further, given the age distribution of our cohort and to determine whether skills continue to be gained across ages (versus stagnation or decline), we also evaluated associations utilizing raw values in addition to the v-scale scores on the Vineland measure subdomains. Across all statistical analyses, significance was set at *p* < 0.050 (two-sided), and trending results were reported as being between 0.050 < *p* < 0.077 (two-sided).

## 3 Results

Our cohort consisted of 71 individuals with RSTS between the ages of one and 61 years, with 36 females (51%) and 35 males (49%) and with 51% of individuals with RSTS1 (Median age = 10.70 years), 27% with RSTS2 (Median age = 8.90 years) and 23% having a clinical diagnosis (Median age = 34.74 years; Table 1, genetic variants in Supplementary Table S1). Four individuals with RSTS1 had reported variants of uncertain significance by the performing laboratory. One individual had subsequent DNA methylation testing consistent with a diagnosis of RSTS1 (c.5051C>A, p. Ser1684Tyr). The second individual was evaluated by an expert geneticist within the RSTS community and has a rare variant predicted to be deleterious within *in silico* models (c.86–3T>G, p.?). The third individual has a previously reported variant in an individual with RSTS and a consistent medical history and dysmorphology examination (c.5933A>G, p. Asn1978Ser) (Coupry et al., 2002). The final individual has a consistent medical history along with a *de novo* variant predicted to be deleterious and reported in multiple other individuals within ClinVar (c.4439A>G (p.D1480G)).

**TABLE 1:** Demographic Characteristics of RSTS Sample.

*Characteristic*	*n*	*%*	*Female/Male*	*M (SD)*	
Age (years)	71			16.89 (13.96)	
Age Group					
Early Childhood (0–5 years)	15	21%	8/7	4.11 (1.19)	
School Age (6–12 years)	22	31%	10/12	9.96 (1.55)	
Adolescence/Early Adulthood (13–21 years)	18	25%	11/7	16.61 (2.87)	
Adulthood (22+ years)	16	23%	7/9	38.74 (12.35)	
Sex					
Female	36	51%			
Male	35	49%			
Race					
White	61	86%			
Black or African American	1	1%			
Asian	2	3%			
Multiple Races	6	9%			
Not Reported	1	1%			
Ethnicity					
Hispanic	8	11%			
Non-Hispanic	61	86%			
Not Reported	2	3%			
Diagnosis					
RSTS1 (*CREBBP)*	36	51%	19/17		
Variant Available	33	92%			
Participant Report	3	8%			
RSTS2 (*EP300)*	19	27%	9/10		
Variant Available	19	100%			
Clinical	16	23%	8/8		
*Measure*	*n*	*Validated Age Range*	*Age Range Completed*	*Age Range Included in Analyses*	*Included n*
Y-BOCS	71	8–18+ years	1–61 years	6–61 years	56
SCAS	71	6–18 years	1–61 years	6–61 years	56
ABC-2	71	5 years-adulthood	1–61 years	5–61 years	59
Vineland	56	Birth-90 years	1–61 years	1–61 years	56

M: mean and SD: standard deviation.

**FIGURE 1:**
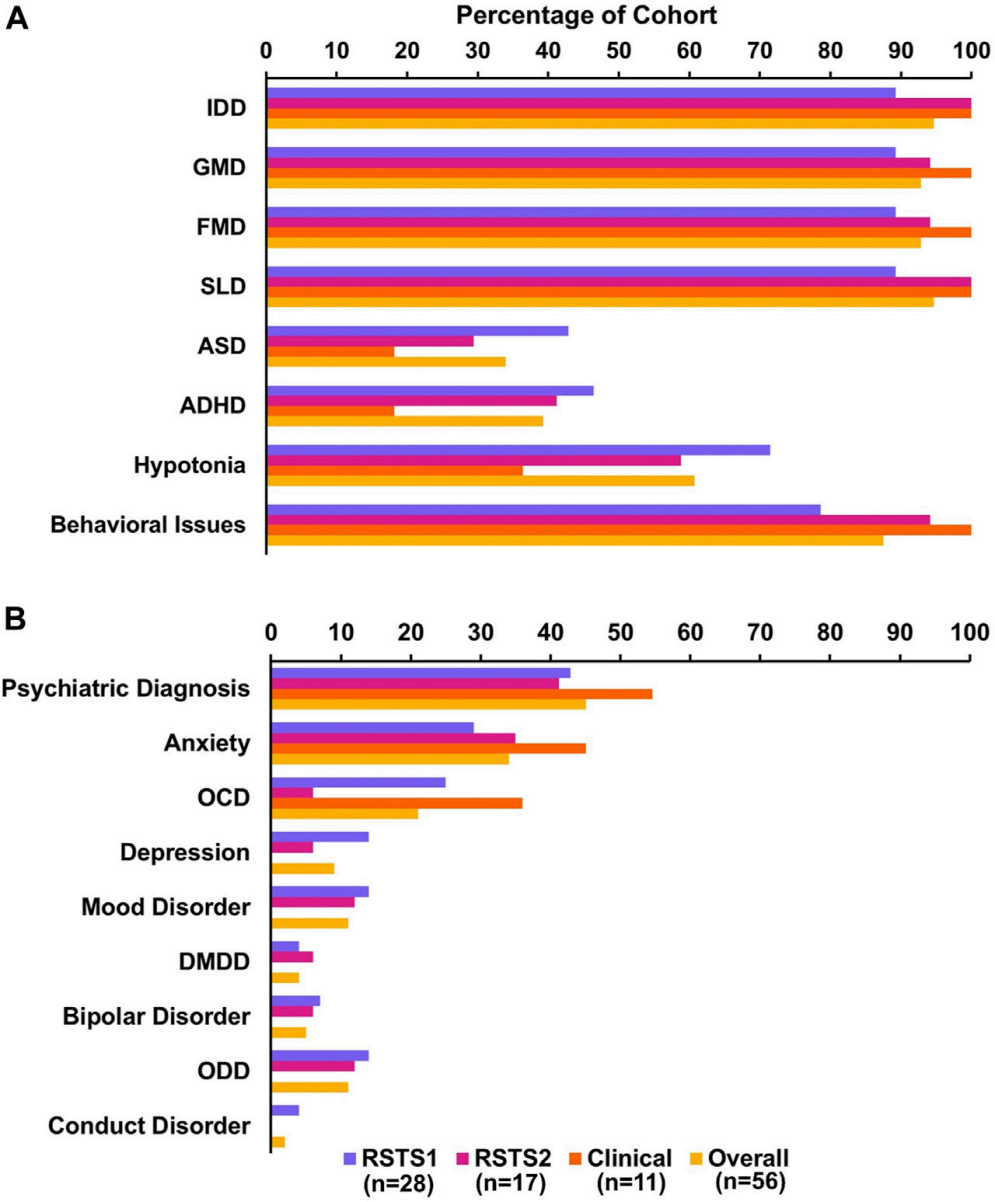
Neurobehavioral Phenotype of RSTS Sample. **(A)** Neuropsychological characteristics represented in our sample including intellectual and developmental disability (IDD), gross motor delay (GMD), fine motor delay (FMD), speech and language delay (SLD), autism spectrum disorder (ASD), and attention deficit hyperactivity disorder (ADHD). **(B)** Breakdown of psychiatric diagnoses in our sample including obsessive compulsive disorder (OCD), disruptive mood dysregulation disorder (DMDD), and oppositional defiant disorder (ODD).

Our cohort was representative across age groups with 21% of individuals in early childhood (0–5 years), 31% of school age individuals (6–12 years), 25% of adolescent/early adulthood individuals (13–21 years) and 23% of adult age individuals (22 years and older). Within the early childhood group, 2 individuals were between 1 and 2 years of age, 4 between 3 and 4 years, 6 between 4 and 5 years and 3 between 5 and 6 years of age. Ninety-five percent of individuals reported intellectual and developmental disability (IDD) with 93%–95% reporting some domain of developmental delay. Further, 34% overall reported a diagnosis of autism spectrum, and 39% reported attention deficit hyperactivity disorder. Forty-five percent reported a psychiatric diagnosis with 34% reporting anxiety, 21% reporting OCD, 9% reporting depression, 11% reporting mood disorders, 4% reporting disruptive mood dysregulation disorder (DMDD), 5% reporting a diagnosis of bipolar disorder, 11% reporting oppositional defiant disorder (ODD), and 2% reporting conduct disorder. Finally, the majority of our cohort reported behavioral issues with 88% of individuals in total (Figure 1; Supplementary Table S2).

### 3.1 Overall cohort

OCD symptoms were common with 82% of the total cohort having total Y-BOCS scores above the subclinical range. A large percentage showed anxious symptoms with individuals with RSTS scoring above threshold on SCAS subscales of panic attack and agoraphobia (52%), separation anxiety (45%), physical injury fears (46%), social phobia (25%), obsessive compulsive (39%), and GAD/overanxious disorder (32%) (Whiteside & Brown, 2008). Our sample also appears to have overall higher challenging behaviors compared to individuals with intellectual disability, especially within individuals younger than 14 years (Brown et al., 2002) (Supplementary Table S3). Of note, these data only reflect scores from individuals above the validated age range for the questionnaires, see Methods. A majority of our cohort (45%–60%) fell below the first percentile on the Adaptive Behavior Composite and all three domains of the Vineland (Supplementary Table S3). Further, comparison to scores in developmentally delayed peers (2–9 years) showed lower average scores on the Adaptive Behavior Composite as well as all domains and subdomains of the Vineland, meaning our 2–9 year old participants are above the threshold for a diagnosis of developmental delay as determined by the Vineland (Sparrow et al., 2016). This pattern was also evident when extending this comparison to our entire sample (Supplementary Table S3).

### 3.2 Age-related differences

A majority of individuals after early childhood had increased OCD symptoms with: 86% of school age, 83% of adolescence/early adulthood, and 75% of adulthood groups with total Y-BOCS scores above the subclinical range (Supplementary Table S3). Comparison to normative GAD/overanxious disorder subscale scores revealed 27% of school age individuals, 33% of adolescent/early adulthood individuals and 38% of those in adulthood reported mean subscale scores higher than general population norms (Supplementary Table S3). When evaluating Y-BOCS and SCAS measures across ages, no significant results were seen across ages (Table 2). When evaluating the full cohort with younger individuals included, it appears that obsessive compulsive and GAD/overanxious behaviors may increase over time.

**TABLE 2:** Neuropsychiatric and Behavioral Abilities and Challenges over RSTS Age Groups.

Measure		Overal M(SD)	Early Childhood^a^ M(SD)		School Age^b^ M(SD)	Adolescence/Early Adulthood^c^ M(SD)	Adulthood^d^ M(SD)	F	p	Post-hoc
*Y-BOCS*	n	56		15	22	18	16			
	Obsessive Thoughts	7.82 (5.12)		3.99 (4.25)	6.95 (5.71)	8.35 (4.68)	8.41 (4.88)	0.51	0.605	
	Compulsive Behaviors	8.46 (5.68)		3.58 (4.09)	8.06 (6.00)	8.80 (5.30)	8.63 (5.98)	0.09	0.914	
	Total	16.65 (9.84)		7.81 (7.82)	15.68 (10.20)	17.46 (9.03)	17.06 (10.70)	0.18	0.840	
*SCAS*	n	56		15	22	18	16			
	Panic Attack and Agoraphobia	2.89 (3.37)		2.20 (2.88)	2.86 (3.60)	2.33 (2.45)	3.56 (3.97)	0.56	0.576	
	Separation Anxiety	3.18 (3.03)		2.73 (2.52)	3.50 (3.25)	2.72 (2.20)	3.25 (3.61)	0.325	0.724	
	Physical Injury Fears	3.20 (2.71)		2.27 (2.15)	2.95 (2.77)	3.22 (2.73)	3.50 (2.76)	0.18	0.833	
	Social Phobia	2.46 (3.42)		1.57 (1.79)	1.64 (3.16)	3.17 (3.20)	2.81 (3.94)	1.11	0.336	
	Obsessive Compulsive	3.11 (3.20)		0.79 (1.19)	2.95 (3.40)	2.89 (2.37)	3.56 (3.83)	0.22	0.801	
	GAD/Overanxious Disorder	3.57 (2.80)		1.50 (2.18)	3.05 (2.90)	3.78 (2.53)	4.06 (3.00)	0.67	0.514	
*ABC-2†*	n	59		3	22	18	16			
	Irritability	10.32 (8.97)		6.33 (5.51)	14.27 (11.02)	7.61 (5.79)	8.69 (7.84)	2.53	0.067	
	Social Withdrawal	9.29 (8.59)		8.33 (3.51)	11.91 (10.14)	7.83 (7.41)	7.50 (7.85)	1.10	0.356	
	Stereotypic Behavior	5.36 (4.94)		3.67 (2.31)	7.96 (6.09)	3.73 (3.71)	3.95 (3.19)	3.71	0.017	b>c**,d*
	Hyperactivity/Noncompliance	13.93 (10.37)		14.67 (12.66)	21.68 (10.48)	9.39 (5.71)	8.25 (7.52)	10.06	<0.001	b>c***,d***
	Inappropriate Speech	3.10 (3.16)		2.67 (2.08)	3.32 (3.17)	3.22 (3.35)	2.75 (3.32)	0.12	0.947	
*Vineland Standard and Subdomain V-scale Scores*	n	56		14	18	14	10			
	ABC	58.27 (16.16)		64.36 (7.87)	59.67 (17.77)	61.21 (16.04)	43.10 (14.39)	4.53	0.007	d < a**, b**, c**
	Communication	56.54 (19.85)		60.57 (14.33)	56.44 (22.59)	64.21 (17.08)	40.30 (17.77)	3.56	0.020	d < a*, b*, c**
	Receptive	7.66 (3.67)		7.79 (3.12)	7.17 (4.66)	8.57 (2.95)	7.10 (3.54)	0.470	0.708	
	Expressive	8.21 (4.25)		6.57 (3.84)	7.94 (4.93)	10.00 (3.88)	8.50 (3.50)	1.61	0.198	
	Written	6.83 (3.70)		8.58 (3.34)	6.50 (3.24)	8.00 (3.44)	3.70 (3.56)	4.57	0.007	d < a**, b*, c**
	Daily Living Skills	53.95 (17.63)		58.29 (14.14)	55.94 (17.10)	57.36 (19.57)	39.50 (14.75)	3.09	0.035	d < a**, b*, c*
	Personal	6.34 (3.66)		5.79 (2.89)	6.17 (3.88)	7.50 (4.45)	5.80 (3.12)	0.650	0.587	
	Domestic	7.17 (3.37)		8.42 (2.68)	7.67 (3.09)	7.50 (3.41)	4.30 (3.37)	3.61	0.020	d < a**, b**, c*
	Community	6.35 (3.40)		7.83 (2.37)	6.39 (3.50)	6.86 (3.70)	3.80 (2.78)	3.06	0.036	d < a**, b*, c*
	Socialization	59.14 (19.96)		69.50 (10.70)	63.11 (21.93)	58.86 (18.65)	37.90 (12.55)	7.00	<0.001	d < a***, b***, c**
	IP Relationships	7.85 (3.62)		8.43 (1.87)	8.33 (4.30)	8.00 (4.21)	6.00 (3.13)	1.11	0.353	
	Play and Leisure	7.88 (3.46)		9.00 (2.75)	8.11 (4.16)	8.07 (3.43)	5.60 (2.22)	2.09	0.113	
	Coping Skills	8.11 (3.20)		10.31 (1.89)	8.11 (3.77)	7.29 (3.05)	6.40 (2.27)	3.82	0.015	a > b*, c*, d**

F and *p*-values represent comparisons between age groups and exclude the early childhood group for the Y-BOCS and SCAS measures. Post-hoc analyses represent exclusion of early childhood group for the Y-BOCS and SCAS measures. Asterisks represent statistical significance (*p < 0.05; **p < 0.01; ***p < 0.001). M: mean, SD: standard deviation, GAD: generalized anxiety disorder, ABC: adaptive behavior composite, and IP: interpersonal. † - the “Early Childhood” group only refers to the 5-year-old participants for the ABC-2 measure given validated range beginning at 5 years.

**FIGURE 2:**
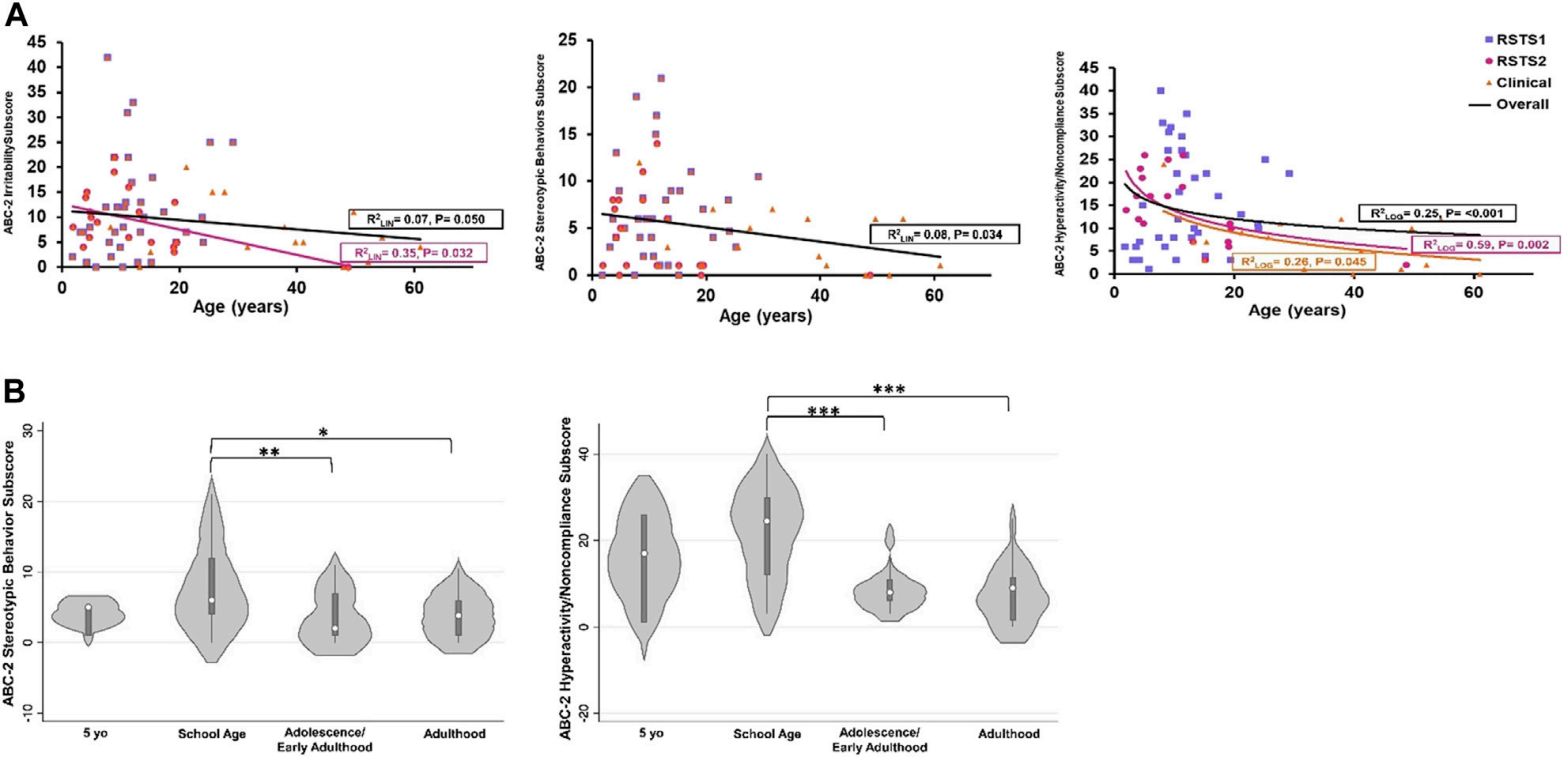
Challenging Behaviors across Age and between Age Groups. **(A)** Stereotypic behavior decreases across age. All data points are included for data visualization, but age regression analysis was performed with only validated ages. **(B)** Stereotypic behaviors and hyperactivity/noncompliance are highest in school age children. Asterisks represent statistical significance (*p < 0.050; **p < 0.010; ***p < 0.001).

Challenging behaviors differed across ages within the irritability (r_lin_^2^ = 0.07, *p*-value = 0.050), stereotypic behaviors (r_lin_^2^ = 0.08, *p*-value = 0.034), and hyperactivity/noncompliance (r_log_^2^ = 0.25, *p*-value= <0.001) subscales (Figure 2A). Challenging behaviors were reported at higher rates in younger individuals including within scores for stereotypic behaviors (F_(3,55)_ = 3.71, *p*-value = 0.017, η^2^ *p* = .17) and hyperactivity/noncompliance (F_(3, 55)_ = 10.06, *p*-value<0.001, η^2^ *p* = .35). School age children had significantly higher ratings than adolescents/early adults (t-values >2.88, *p*-values<0.006) and adults (t-values>2.64, *p*-values<.011) (Table 2; Figure 2B). Irritability was marginally higher as well (F_(3,55)_ = 2.53, *p*-value = 0.067, η^2^ *p* = .12). Findings remained consistent when analyzing the entire cohort.

**FIGURE 3:**
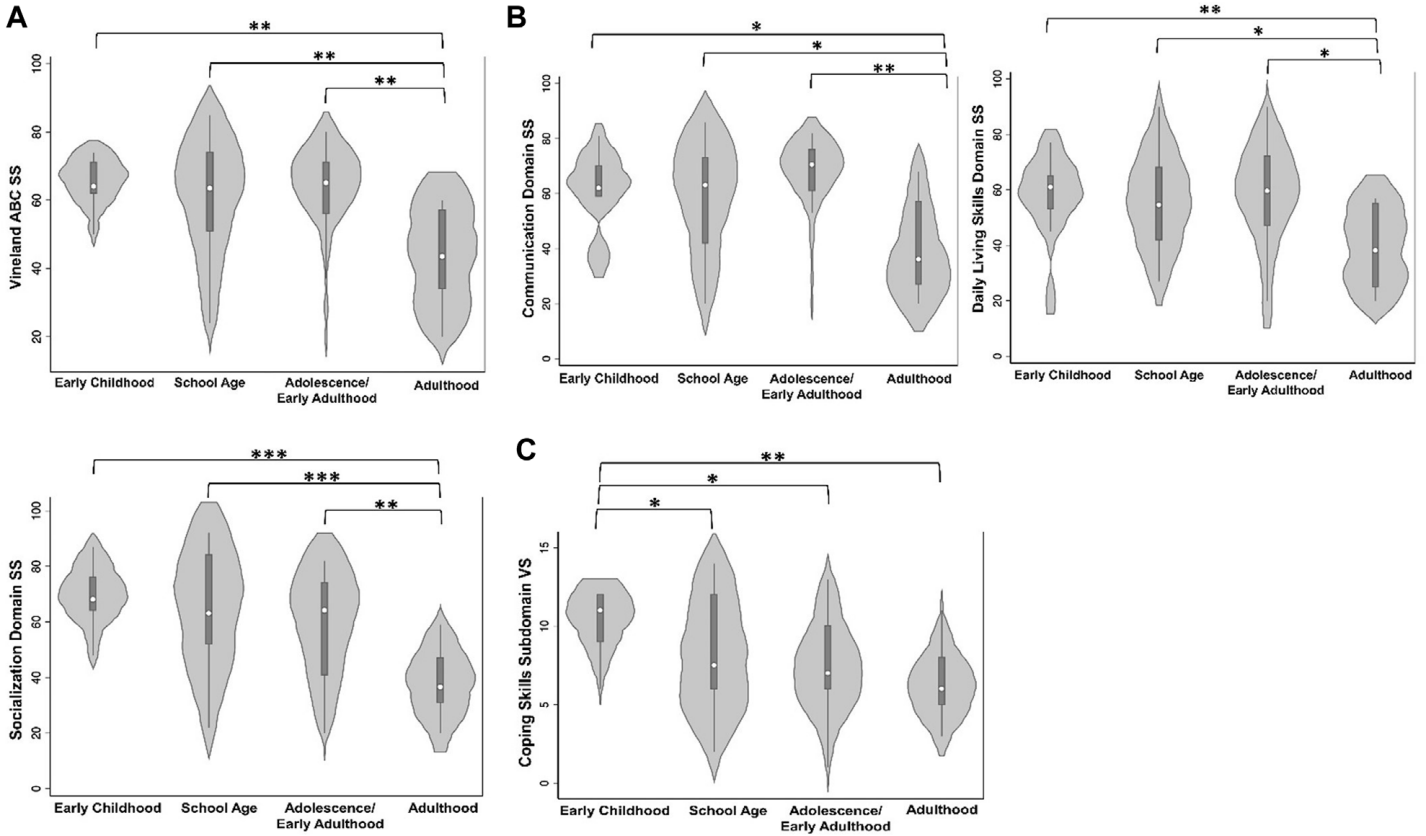
Adaptive Behavior and Living Skills between Age Groups. **(A)** Adaptive Behavior Composite standard scores (SS) are lowest in adults. **(B)** Communication and Daily Living Skills domain SS are lowest in adults. **(C)** Coping skills subdomain v-scale scores (VS.) are lower in individuals of school age, adolescence/early adulthood, and adulthood compared to early childhood individuals. Asterisks represent statistical significance (*p < 0.050; **p < 0.010; ***p < 0.001).

Differences in adaptive behavior and living skills were significant across ages. The Adaptive Behavior Composite (F_(3, 52)_ = 4.53, *p* = 0.007, η^2^ *p* = .21) and domain scores (F_(3, 52)_>3.09, *p*-values<0.035, η^2^ *p*-values>.15) were different between age groups with individuals in adulthood reporting lower standard scores compared to all younger groups (t-value’s > 2.00, *p*-values<0.032; Table 2; Figures 3A,B). Further, Written (F_(3, 51)_ = 4.57, *p*-value = 0.007, η^2^ *p* = .22), Domestic (F_(3, 51)_ = 3.61, *p*-value = 0.020, η^2^ *p* = .18), and Community (F_(3, 51)_ = 3.06, *p*-value = 0.036, η^2^ *p* = .16) subdomain v-scale scores were significantly different across ages with lower scaled scores being reported in adulthood compared to all other age groups (t-values>2.04, *p*-values<0.047) (Table 2). Additionally, v-scale scores in the Coping Skills subdomain also differed between age groups (F_(3, 51)_ = 3.82, *p*-value = 0.015, η^2^ *p* = .18), with individuals in the early childhood age group having higher scaled scores than all older age groups (t-values>2.03, *p*-values<0.048) (Table 2; Figure 3C).

**FIGURE 4:**
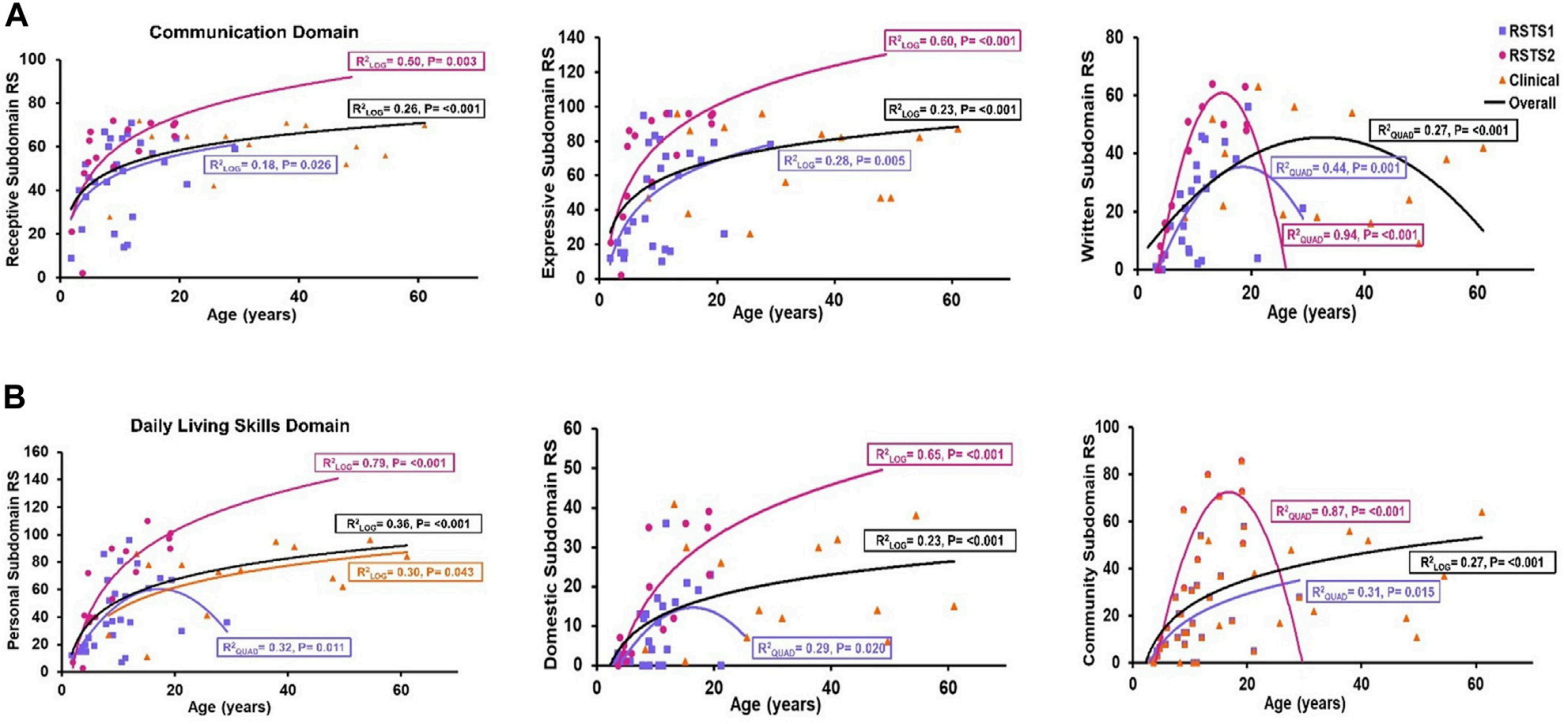
Communication and Daily Living Skills across Age. **(A)** Receptive and Expressive language raw scores (RS) are higher among individuals of older age while written language skills were lower in older ages. **(B)** Personal, Domestic, and Community skills RS increase across age. The black trendline represents the overall trend across all 3 cohorts.

Although examination of raw scores in different age groups (Supplementary Table S5) seem to suggest potential stagnation of adaptive skills in adulthood, exploring best-fit regression models across all ages indicates individuals with RSTS continue to gain skills across ages in areas of Receptive (r_log_^2^ = 0.26, *p*-value<0.001) and Expressive Communication subdomains (r_log_^2^ = 0.23, *p*-value<0.001), all Daily Living Skills subdomains (r_log_^2^ = 0.23–0.36, *p*-values≤0.001), and all Socialization subdomains (r_log_^2^ = 0.10–0.11, *p*-values<0.022) (Supplementary Table S4; Figure 4). In contrast, raw scores in the Written subdomain demonstrated peak in early adulthood followed by a subsequent decline (r_quad_^2^ = 0.27, *p*-value<0.001) (Supplementary Table S4; Figure 4), consistent with findings from comparisons across age groups in which raw scores were lower in the adult group than the adolescence/early adulthood group (t-value = 2.28, *p*-value = 0.021; Supplementary Table S5). With the exception of Coping Skills, all subdomain Growth Scale Values (GSVs) are higher across ages from early childhood to early adulthood, then show stagnation in adulthood.

### 3.3 Sex

In contrast to the general population, both sexes reported similar total Y-BOCS scores above the subclinical threshold (82%; Supplementary Table S3), compared to the typically increased symptomatology reported in males. Comparison to normative GAD/overanxious disorder subscale scores revealed 29% of male individuals and 36% of female individuals reported mean subscale scores higher than general population norms (Supplementary Table S3). Despite our anecdotal observation of more behavioral concerns reported from caregivers of adolescent and adult females with RSTS than from caregivers of adolescent and adult males, we found no significant overall sex-related differences on any measure including the Y-BOCS, SCAS, ABC-2 and across Vineland domain scores and subdomain scores (F-values_(1,53)_ < 1.80, *p*-values > 0.185; Supplementary Table S6). However, we did observe differences based on sex within our type analyses, discussed below.

### 3.4 RSTS1 to RSTS2 type comparisons

OCD symptomatology above the subclinical threshold showed overall even distribution and high prevalence among RSTS type with 90% of individuals with RSTS1, 73% of individuals with RSTS2, and 75% of individuals with a clinical diagnosis showing elevated total Y-BOCS scores when excluding individuals in the early childhood age group (Supplementary Table S3). The GAD/overanxious disorder subscale on the SCAS revealed 28% of individuals with RSTS1, 45% of individuals with RSTS2, and 31% of individuals with clinical RSTS reported mean subscale scores above the general population norms when excluding individuals in the early childhood age group (Supplementary Table S3).

**FIGURE 5:**
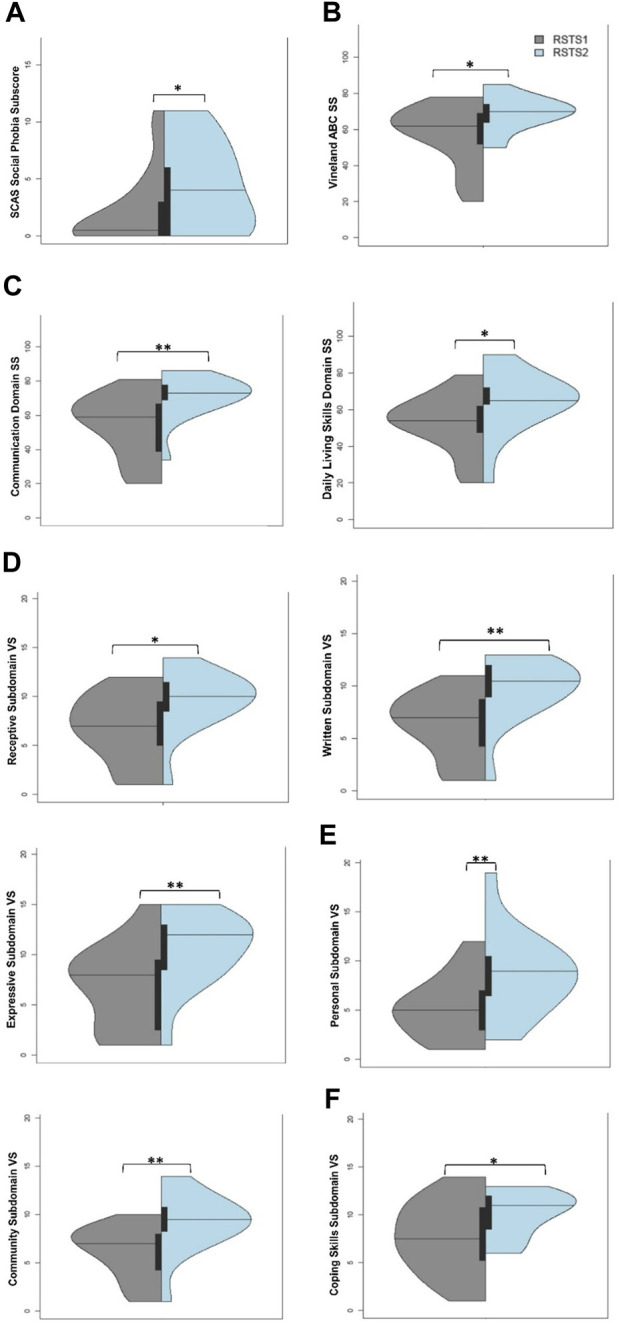
Adaptive Behavior and Living Skills between Individuals with RSTS1 and RSTS2. **(A)** Individuals with RSTS2 have higher social phobia than individuals with RSTS1. **(B–C)** Adaptive Behavior Composite and Communication and Daily Living Skills domain standard scores (SS) were lower in individuals with RSTS1. **(D–F)** Across the depicted subdomains, v-scale scores (VS.) were lower in individuals with RSTS1. Asterisks represent statistical significance (*p < 0.05; **p < 0.01; ***p < 0.001).

No significant differences between individuals with RSTS1 and RSTS2 were seen in obsessive compulsive or anxiety symptoms based on the Y-BOCS and SCAS scores (F-values_(2,68)_<2.47, *p*-values>0.092, η^2^ *p*-values<.068) with the exception of social phobia. Individuals with RSTS2 had higher social phobia ratings than individuals with RSTS1 (F_2,53_ = 3.75, *p* = 0.030, η^2^ *p* = .12; t-value = 2.37, *p*-value = 0.022) (Table 3; Figure 5A). When assessing challenging behaviors, a significant difference between types emerged for stereotypic behavior (F_2,56_ = 3.24, *p* = 0.046, η^2^ *p* = .10) with individuals with RSTS1 having marginally higher ratings than individuals with RSTS2 (t-value = 1.94, *p*-value = 0.057). Supplementary Table S7. When including the entire cohort, this finding became significant (F_2,68_ = 3.349, *p* = 0.041, η^2^ *p* = .09; t-value = 2.01, *p* = 0.049).

**TABLE 3:** Neuropsychiatric and Behavioral Abilities and Challenges by RSTS Type.

Measure		RSTS1^a^ M (SD)	RSTS2^b^ M (SD)	F	*p*	Post-hoc
*Y-BOCS*	n	29	11			
	Obsessive thoughts	8.02 (5.67)	8.55 (3.86)	0.35	0.706	
	Compulsive behaviors	9.84 (6.08)	6.73 (5.35)	1.85	0.168	
	Total	18.56 (10.33)	15.27 (8.82)	1.19	0.313	
*SCAS*	n	29	11			
	Panic attack and agoraphobia	3.28 (4.02)	3.09 (2.81)	0.69	0.508	
	Separation anxiety	3.34 (3.38)	3.27 (2.69)	0.16	0.852	
	Physical injury fears	2.97 (2.46)	4.36 (3.80)	1.30	0.282	
	Social phobia	2.10 (3.23)	4.82 (4.14)	3.75	0.030	a < b*
	Obsessive compulsive	3.62 (3.64)	2.64 (2.50)	0.77	0.466	
	GAD/overanxious disorder	3.34 (3.04)	4.64 (2.54)	0.99	0.377	
*ABC-2*	n	30	13			
	Irritability	12.67 (10.55)	9.46 (6.58)	2.58	0.085	
	Social withdrawal	9.83 (9.01)	10.77 (8.73)	0.78	0.462	
	Stereotypic behavior	6.91 (5.35)	3.85 (4.62)	3.24	0.046	
	Hyperactivity	17.80 (11.04)	13.54 (8.67)	6.80	0.002	
	Inapp. speech	3.93 (3.56)	1.77 (2.24)	2.50	0.091	
*Vineland standard and subdomain V-scale scores*	n	27	15			
	ABC	57.19 (16.73)	68.80 (9.64)	6.65	0.003	b > a*
	Communication	53.00 (19.00)	71.27 (12.56)	7.37	0.001	b > a***
	Receptive	6.70 (3.61)	9.67 (3.04)	3.50	0.037	b > a*
	Expressive	6.67 (4.27)	10.53 (3.62)	4.66	0.014	b > a**
	Written	6.19 (3.14)	9.71 (3.07)	7.62	0.001	b > a**
	Daily living skills	52.96 (15.52)	64.87 (17.12)	6.01	0.004	b > a*
	Personal	5.30 (3.21)	8.53 (4.27)	4.32	0.018	b > a**
	Domestic	7.08 (2.86)	8.86 (3.16)	3.51	0.037	
	Community	6.00 (2.76)	9.21 (3.09)	11.15	<0.001	b > a**
	Socialization	60.81 (21.82)	69.87 (12.93)	7.50	0.001	
	IP relationships	7.70 (3.84)	9.47 (3.34)	2.76	0.072	
	Play and leisure	7.96 (3.90)	9.20 (2.88)	2.74	0.074	
	Coping skills	7.73 (3.74)	10.07 (2.34)	4.95	0.011	b > a*

Analyses exclude the early childhood group for the Y-BOCS and SCAS measures. Analyses include only 5-year-old children from the early childhood group for the ABC-2 measure. Asterisks represent statistical significance (* *p* < 0.05; ** *p* < 0.01; *** *p* < 0.001). M, mean; SD, standard deviation; ABC, adaptive behavior composite; IP, interpersonal.

Significant differences were seen upon evaluation of adaptive behavior and living skills. The Adaptive Behavior Composite (F_2,53_ = 6.65, *p*-value = 0.003, η^2^ *p* = .20) as well as Communication (F_2,53_ = 7.37, *p*-value = 0.001, η^2^ *p* = .22) and Daily Living Skills (F_2,53_ = 6.01, *p*-value = 0.004, η^2^ *p* = .19) domain standard scores were significantly higher in individuals with RSTS2 compared to individuals with RSTS1 (Table 3; Figures 5B,C; t-values>2.28, *p*-values<0.027). A similar pattern emerged across nearly all subdomain v-scale scores in which individuals with RSTS2 had significantly higher scores than those with RSTS1 (F_(2,53)_’s > 3.50, *p*-values<0.037, η^2^ *p*-values>0.12; Table 3; Figures 5D,E). While the Socialization domain was not significant overall, individuals with RSTS2 had higher scaled scores within the Coping Skills subdomain than individuals with RSTS1 (t-value = 2.08, *p* = 0.042; Figure 5F). However, only marginal differences were noted within the Interpersonal Relationships and Play and Leisure subdomains (Table 3; F_2,53_ > 2.74, *p*-value<0.074, η^2^ *p*-values = 0.09) (Supplementary Table S7).

**FIGURE 6:**
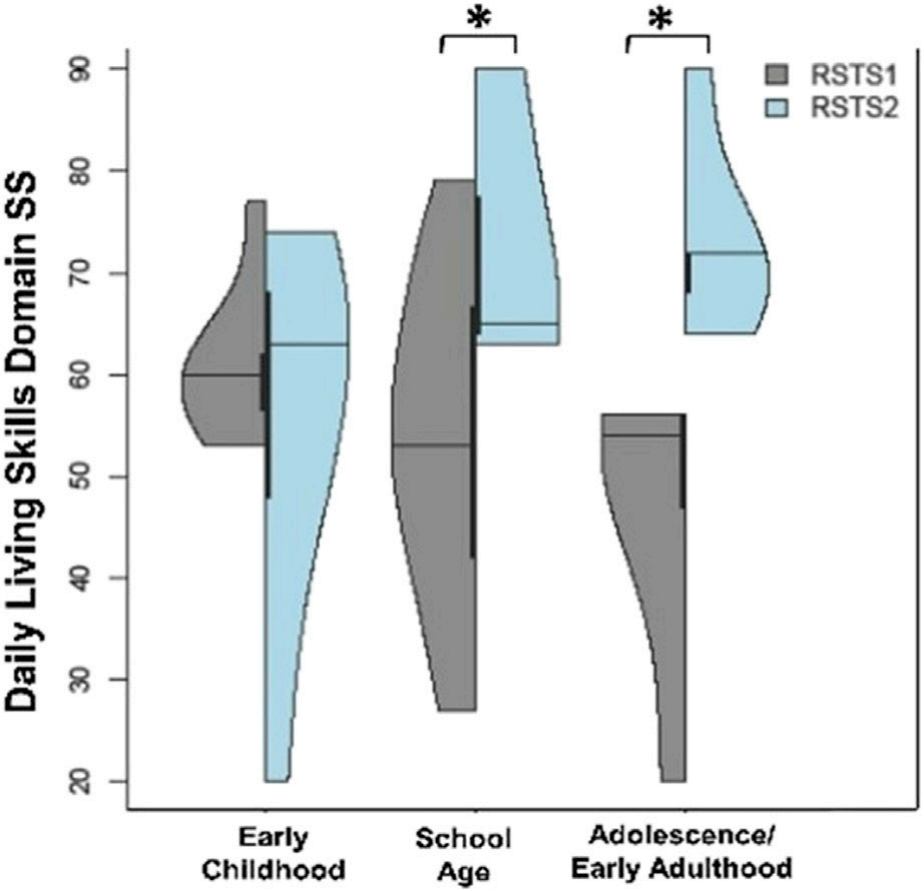
Daily Living Skills between RSTS Types and Based on Age. Individuals with RSTS2 have higher daily living skills between school age and adolescence/early adulthood than individuals with RSTS1 of the same age. Asterisks represent statistical significance (*p < 0.05; **p < 0.01; ***p < 0.001).

Given the significant differences reported by RSTS type and over ages, we assessed if type differences may be present at certain ages. Overall, no significant findings were seen within the Y-BOCS measure. However, given our *a priori* clinical observations, we assessed if any age-related differences may exist by type. There was a marginal finding of adults with RSTS1 having higher obsessions than school age children with RSTS1 (t-value = 2.00, *p* = .0.054) as well as possibly higher compulsions than adults with RSTS2, but this observation is limited due to sample size (t = 2.12, *p* = 0.042). No other findings were notable. On anxiety measures, despite differing levels of social phobia scores between types, no age-related differences by type were seen. For challenging behaviors, no significant RSTS type differences by age were seen for stereotypic behaviors, irritability or hyperactivity. However, the higher scores within school age individuals seen overall may be driven by individuals with RSTS2 given these individuals may have higher scores than adolescents/EA individuals with RSTS2 (stereotypic behaviors: t-value = 2.27, *p*-value = 0.027; hyperactivity: t-value = 2.62, *p*-value = 0.012). When assessing adaptive behavior and living skills, Daily Living Skills domain scores were different between RSTS types across ages with higher scores in individuals with RSTS2 between school age and adolescence/early adulthood when compared to individuals with RSTS1 of the same age (t-value>2.05, *p*-values<0.048; Figure 6) with subdomain scores aligning with these findings. Community domain scores were also higher in school age individuals with RSTS2 compared to school age individuals with RSTS1 (t-value = 2.21, *p*-values = 0.034) with only marginal differences seen between adolescents/early adulthood individuals (t-value = 1.92, *p* = 0.063).

**FIGURE 7:**
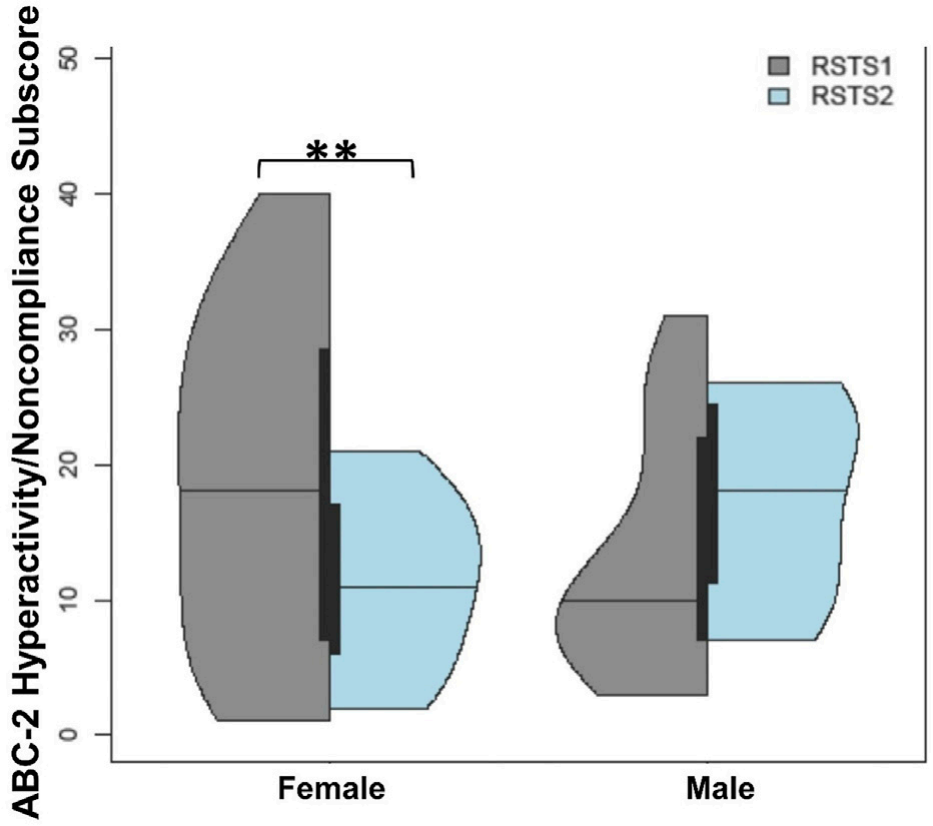
Challenging Behaviors between RSTS Types and Based on Sex. Females with RSTS1 experienced more hyperactivity/noncompliance than females with RSTS2. Asterisks represent statistical significance (**p < 0.01).

Last, we examined whether sex differences differed by RSTS type. No differences were seen between RSTS type and sex within the Y-BOCS or SCAS measures. A pattern of findings emerged for hyperactivity/noncompliance (F_(2,53)_ = 3.45, *p*-value = 0.039, η^2^ *p* = .12) with female individuals with RSTS1 having higher hyperactivity/noncompliance scores than female individuals with RSTS2 (t-value = 2.78, *p*-value = 0.008; Figure 7) and marginally higher scores than males with RSTS1 (t-value = 1.81, *p*-value = 0.076). Interestingly, males with RSTS2 also showed marginally higher rates than females with RSTS2 (t-value = 1.85, *p*-value = 0.070).

## 4 Discussion

Our paper systematically examines neuropsychiatric and behavioral challenges across ages and between types and sexes in one of the largest cohorts of individuals with RSTS. We report four key findings. First, neuropsychiatric and behavioral challenges are prevalent within individuals with RSTS especially in early life. Second, school age behavioral elevations were observed in irritability, stereotypic behaviors, and hyperactivity/noncompliance. Third, individuals with RSTS2 displayed higher scaled scores on adaptive behavior and living skills compared to individuals with RSTS1. Finally, type-related sex differences may exist within hyperactivity.

### 4.1 Individuals with RSTS have increased neuropsychiatric and behavioral challenges

Neuropsychiatric and behavioral concerns were present in our cohort at high rates. When assessing OCD symptomatology, rates were prevalent. Although 1%–3% of the general population has a diagnosis of OCD (Valleni-Basile et al., 1994; Weissman et al., 1994; Kessler et al., 2005), 21% of individuals in our sample reported an OCD diagnosis, and 82% had mild to severe OCD-like symptoms as indicated by the Y-BOCS, similar to prior studies (Levitas & Reid, 1998; Stevens et al., 2011), though at an increased rate. However, past reports have been mixed with a small study of 27 individuals showing lower scores on the obsessive compulsive subscale of the SCAS compared to children diagnosed with OCD (Crawford et al., 2017).

Similar to OCD-like symptoms, anxiety symptoms were commonly reported in our cohort with above normative values on the SCAS, consistent with previous reports of increased anxiety in RSTS (Gotts & Liemohn, 1977; Whiteside & Brown, 2008; Crawford et al., 2017). However, despite increased symptomology on validated measures, only 34% of our cohort reported a diagnosis of anxiety. Past reports are conflicting on anxious symptoms as one study within younger individuals (mean = 8 years) showed lower levels of anxiety compared to typically developing children (Galéra et al., 2009). Social phobia appears to be a less impacted area of anxiety overall as only 25% of individuals in our sample reported scores above average compared to normative data available. Despite this, we found social phobia is elevated within individuals with RSTS2 compared to RSTS1. One past study reported lower levels of social phobia when compared to typically developing individuals (Crawford et al., 2017). However, within that study, individuals with RSTS showed increased levels of panic attack and agoraphobia and obsessive compulsive symptoms and similar levels of physical injury fears and GAD/overanxious disorder without type subgroup analyses (Crawford et al., 2017).

Behavioral issues were reported in 88% of our sample upon subjective history reporting questions, which was corroborated upon validated measures, consistent with a prior study utilizing validated assessments (Galéra et al., 2009), with significant challenging behaviors seen especially in childhood ages. Further, 28% were currently taking a drug with behavioral or psychological indications (Supplementary Table S9). Of note, historically, behavioral challenges have been reported within a wide range (10%–76%) in individuals with RSTS (Stevens et al., 1990; Boer et al., 1999; Wincent et al., 2016).

Adaptive behavior and living skills overall were noted to fall within lower ranges when compared to scores based on normative data with the majority falling lower than the fifth percentile for age on all scores of the Vineland. This is consistent with a prior study assessing receptive and expressive speech development as these areas of development were lower than typically developing children (Adrien et al., 2021).

### 4.2 Individuals with RSTS have changing neuropsychiatric and behavioral challenges across ages

We observed differences in behaviors across age of individuals with RSTS. Irritability, stereotypic behaviors, and hyperactivity/noncompliance appear to peak in our school age cohort, consistent with prior studies. Galéra et al. (2009) showed stereotypies were elevated in individuals with RSTS within a cohort with an average age of 8 years (range: 4–15 years). Further, apparent age-related behavior is consistent with emotion dysregulation peaking during this age span in other genetic syndromes associated with developmental delay and intellectual disability (Shaffer et al., 2022).

Despite an overall increased prevalence of OCD-like and generalized anxiety symptoms, scores within this area do not differ across age when only including individuals above the validated age range in typically developing children. Our findings are in contrast to those of a prior study revealing a trend in older individuals to report higher scores on the obsessive compulsive subscale of the SCAS (Crawford et al., 2017). However, obsessive compulsive behaviors have not been well-characterized or studied previously within this population. Therefore, OCD within individuals with RSTS is an area that needs further investigation given its prevalence with self-report measures and our anecdotal clinical experience. When considering anxiety, one study comparing individuals with RSTS (mean age of 15.8 years) to typically developing children revealed lower levels of anxiety were reported on the CBCL (Galéra et al., 2009). This is in contrast to findings that anxiety measures increased from infancy to adolescence (0–17 years) with individuals over age 14 years scoring higher on anxiety measures (Yagihashi et al., 2012; Giani et al., 2022).

Individuals with RSTS in our cohort typically scored in the very low range for adaptive skills which corresponds to >90% having an intellectual disability diagnosis. Adults with RSTS tended to have even lower scaled scores on the Vineland than younger individuals, consistent with other neurodevelopmental disorders. Possible reasons for this are numerous. First, adaptive behavior and living skills increases can correspond to cognitive skill increases, which are known to stagnate or decline relative to peers in other genetic syndromes associated with developmental disability (Fisch et al., 2002; Carr, 2005; Hooper et al., 2013). However, and importantly, despite age-related differences between groups on scaled scoring, individuals continue to show improved scores across ages until adulthood as exemplified by raw score assessment. Further, individuals at younger ages have relatively fewer expectations that those in adulthood, also potentially contributing to the widened gap between typically developing adult peers. Second, skills that require practice such as writing skills may deteriorate over time if not continually practiced and/or emphasized, especially once the individuals are no longer in school. Coping skills were also significantly lower across older ages, which has not been reported in past studies. Those studies utilizing the Vineland questionnaire do not report summary scores on the measures, thus direct comparison is not possible at this time.

Individuals with RSTS are known, in part, by their lovely, friendly personalities with validated measures showing higher levels of sociability overall compared to typically developing children (Galéra et al., 2009). However, our findings showed that socialization was significantly lower in adults than all younger age groups. Ellis et al. (2020) found that social motivation does not appear to be compromised in individuals with RSTS, so while the social drive may remain high throughout all ages in RSTS, certain behaviors are more socially acceptable during childhood than in adulthood (e.g., seeking caregiver for comfort), where these behaviors may be considered less typical and may contribute to decreased opportunities for socialization with similar-age peers in older individuals.

### 4.3 Neuropsychiatric and behavioral differences exist between individuals with RSTS1 and RSTS2

Individuals with RSTS2 represented 27% of our sample versus a typical frequency of 8%–10% in the total RSTS population, allowing increased ability to differentiate between the two types of RSTS, which has not been assessed in prior research studies. Previously, type comparison has been limited to subjective reports of autism and autistic-like features with higher reporting of these behaviors in individuals with RSTS1 compared to RSTS2 (Fergelot et al., 2016).

Individuals with RSTS2 reported higher levels of social phobia compared to individuals with RSTS1 without age-related differences seen. Further, with the exception of the Socialization domain, adaptive behavior and living skills scores overall were higher in individuals with RSTS2, which may be related to historically reported higher developmental and intellectual scores in these individuals (Negri et al., 2015; Fergelot et al., 2016; Cohen et al., 2020). Further, type differences were seen within stereotypic behaviors with marginally higher scores reported in individuals with RSTS1 compared to individuals with RSTS2, and both groups reported lower stereotypic behaviors across increasing age, consistent with overall findings. Despite absence of OCD-like and anxiety changes overall, increasing obsessive behaviors may be more apparent in older individuals with RSTS1. We also observed RSTS type differences between sexes as females with RSTS1 had more hyperactivity than females with RSTS2 and possibly males with RSTS1.

Given possible differential challenges related to varying neuropsychiatric and behavioral domains, further study is needed in larger, preferably longitudinal cohorts across all ages to further characterize strengths and weaknesses across RSTS types and between sexes.

### 4.4 Clinical implications and recommendations

Our findings have important clinical implications for the care of individuals with RSTS. When discussing challenging behaviors, families can be counseled that individuals may have a school age peak in these behaviors with likely improvement over time in the teenage years and beyond. As mentioned, discrepancies were noted between a clinical diagnosis of OCD and anxiety, which may indicate a paucity of screening or recognition of these conditions by providers, thus this care gap should be addressed. Providers and caregivers should have an increased index of suspicion for OCD-like behaviors and anxiety. Families and providers should be mindful to minimize anxiety-provoking situations, especially with medical care given their overall medical complexity, as well as stress and support the development of coping skills throughout the lifespan. Of note, behavioral deficits and mental health disorders are linked to sleep disturbances and *vice versa* (Sadeh et al., 2002; Chase & Pincus, 2011). Sleep disorders are reported at high rates within the RSTS community, with 62% of adults in a recent study and 64% of our cohort (Douzgou et al., 2022). Thus, screening for sleep difficulties and optimizing this area of life may positively impact the neuropsychiatric profile of individuals, and thus quality of life. This is an area that deserves further study.

Given our results showing globally decreased adaptive behavior and living skills scores relative to age-matched, typically developing individuals, we recommend all individuals with RSTS continue to practice and reinforce important daily living skills to ensure optimal outcomes and the most independence possible. Individuals with RSTS2 appear to have less marked differences within adaptive behavior and living skills compared to individuals with RSTS1, thus families and individuals with RSTS2 can be counseled of a decreased risk.

To date, there are no RSTS-specific interventions or treatments; thus, general population clinical management guidelines can be followed. Our results corroborate and emphasize the importance of therapies directed towards speech, behavior, and daily living skills.

### 4.5 Limitations

Our study is not without limitations. Our investigation relied on cross-sectional data, thus age-related findings may be due to cohort-related effects instead of changes across time/age. Tracking symptoms over time in a longitudinal sample of individuals is critical to replicate present and past findings. Additionally, a majority of caregivers in our study reported behavioral issues, which could indicate a skewed sample toward those looking for more research in an area that was most impactful for them and their loved ones. Next, our findings are primarily based on caregiver report and not always directly from individuals with RSTS which may lead to response bias and/or misrepresentation of phenotypes assessed. This manner of administration was chosen to be consistent with the validation of chosen measures and the wide range of ages and impacts to cognition seen within RSTS (Aman et al., 1985; Nauta et al., 2004; Sparrow et al., 2016). Further, the SCAS, ABC-2 and Vineland are validated for caregiver-report, and we encouraged caregivers to include their loved ones while completing questionnaires whenever possible. Another possible limitation for our study involves the clinical RSTS group, as these individuals were not able to be molecularly confirmed. Record review was consistent with a diagnosis of RSTS; thus, these individuals were included. Availability of genetic testing for RSTS is relatively recent, and many individuals may be unable to obtain this testing given limitations in insurance payor coverage, research funding availability and distance from our medical center (Petrif et al., 1995; Roelfsema et al., 2005; Graf et al., 2013; Grant et al., 2021). However, our group of individuals were recruited through RSTS-specific support groups and expert clinics without a financial incentive. It should be noted our cohort is overall representative of clinical reality and our data is transferable to the physicians and providers caring for this heterogenous population. Further, an estimated 30% of individuals will not have an identifiable variant in either *CREBBP* or *EP300*, thus 23% of the total cohort not having molecularly confirmation is overall consistent with this measure. Another potential limitation includes the lack of data regarding potential modifiers of adaptive behavior and living skills and other measured characteristics such as developmental interventions (i.e., therapies, clinical support, *etc.*) in which a participant may have engaged across their lifespan. For example, older participants may be receiving or have received fewer interventions than younger participants which could account for reduced severity of symptoms in younger-aged participants. Given this possibility, this area of study is important for future investigations to best delineate the natural history of RSTS. Finally, considering a majority of our cohort identifies as Caucasian, the generalizability of our results across ethnicities is also a limitation and calls for additional studies with greater racially and ethnically diverse samples of individuals with RSTS.

### 4.6 Concluding remarks

Our paper is the first to evaluate systematically behavioral challenges across a wide age range and between types and sexes in individuals with RSTS. Consistent with prior studies, neuropsychiatric and behavioral challenges are common in this population. Challenging behaviors appear to peak within school age individuals as older individuals had lower subscale scores. Further, we are the first to demonstrate apparent similarities and differences between individuals with RSTS1 and RSTS2, highlighting more apparent deficiencies in adaptive behavior and living skills and higher reports of stereotypic behaviors within individuals with RSTS1 as well as increased reports of social phobia for individuals with RSTS2. Obsessive compulsive-like behaviors and anxious symptoms may also be elevated in older individuals with RSTS1. With these findings in mind, families, caregivers, and medical professionals should perform anticipatory guidance and implement proper assessments and interventions in a specific manner based on age, and possibly type, for timely recognition and treatment to improve outcomes and quality of life.

## Data Availability

The raw data supporting the conclusion of this article will be made available by the authors, without undue reservation.

## References

[B1] Achenbach T. M. Ruffle T. M. (2000). The child behavior checklist and related forms for assessing behavioral/emotional problems and competencies. Pediatr. Rev. 21, 265–271. 10.1542/pir.21-8-265 10922023

[B64] Adrien J.-L. Taupiac E. Thiébaut E. Paulais M.-A. Van-Gils J. Kaye K. Blanc R. Gattegno M. P. Contejean Y. Michel G. Dean A. Barthélémy C. Lacombe D. (2021). A comparative study of cognitive and socio-emotional development in children with Rubinstein-Taybi syndrome and children with Autism Spectrum Disorder associated with a severe intellectual disability, and in young typically developing children with matched developmental ages. Research in Developmental Disabilities 116, 104029. 10.1016/j.ridd.2021.104029 34271530

[B2] Ajmone P. Avignone S. Gervasini C. Giacobbe A. Monti F. Costantino A. (2018). Rubinstein-Taybi syndrome: New neuroradiological and neuropsychiatric insights from a multidisciplinary approach. Am. J. Med. Genet. 177 (4), 406–415. 10.1002/ajmg.b.32628 29637745

[B3] Al-Qattan M. M. Jarman A. Rafique A. Al-Hassnan Z. N. Al-Qattan H. M. (2019). Rubinstein-Taybi syndrome in a Saudi boy with distinct features and variants in both the CREBBP and EP300 genes: A case report. BMC Med. Genet. 20 (1), 12. 10.1186/s12881-019-0747-5 30635043 PMC6330443

[B4] Aman M. G. Singh N. N. (2017). Aberrant behavior checklist: Community/residential manual. New York: Slosson Educational Publications, Incorporated.

[B5] Aman M. G. Singh N. N. Stewart A. W. Field C. J. (1985). Psychometric characteristics of the aberrant behavior checklist. Am. J. Ment. Defic. 89, 492–502. (0002-9351 (Print)). 3158201

[B6] Baker E. Arpone M. Kraan C. Bui M. Rogers C. Field M. (2020). FMR1 mRNA from full mutation alleles is associated with ABC-C FX scores in males with fragile X syndrome. Sci. Rep. 10 (1), 11701. 10.1038/s41598-020-68465-6 32678152 PMC7367290

[B7] Bartsch O. Schmidt S. Richter M. Morlot S. Seemanová E. Wiebe G. (2005). DNA sequencing of CREBBP demonstrates mutations in 56% of patients with Rubinstein-Taybi syndrome (RSTS) and in another patient with incomplete RSTS. Hum. Genet. 117 (5), 485–493. 10.1007/s00439-005-1331-y 16021471

[B10] Boer H. Langton J. Clarke D. (1999). Development and behaviour in genetic syndromes: Rubinstein-taybi syndrome. J. Appl. Res. Intellect. Disabil. 12 (4), 302–307. 10.1111/j.1468-3148.1999.tb00086.x

[B11] Breuning M. H. Dauwerse H. G. Fugazza G. Saris J. J. Spruit L. Wijnen H. (1993). Rubinstein-Taybi syndrome caused by submicroscopic deletions within 16p13.3. Am. J. Hum. Genet. 52, 249–254. (0002-9297 (Print)). 8430691 PMC1682202

[B12] Brown E. C. Aman M. G. Havercamp S. M. (2002). Factor analysis and norms for parent ratings on the Aberrant Behavior Checklist-Community for young people in special education. Res. Dev. Disabil. 23 (1), 45–60. 10.1016/S0891-4222(01)00091-9 12071395

[B13] Carr J. (2005). Stability and change in cognitive ability over the life span: A comparison of populations with and without down's syndrome. J. Intellect. Disabil. Res. 49, 915–928. (0964-2633 (Print)). 10.1111/j.1365-2788.2005.00735.x 16287480

[B14] Chase R. M. Pincus D. B. (2011). Sleep-related problems in children and adolescents with anxiety disorders. Behav. Sleep. Med. 9 (4), 224–236. 10.1080/15402002.2011.606768 22003976

[B15] Chromik L. Quintin E. Lepage J. Hustyi K. Lightbody A. Reiss A. (2019). The influence of hyperactivity, impulsivity, and attention problems on social functioning in adolescents and young adults with fragile X syndrome. J. Atten. Disord. 23 (2), 181–188. 10.1177/1087054715571739 25731183 PMC5758425

[B16] Cohen J. Schrier Vergano S. Mazzola S. Strong A. Keena B. McDougall C. (2020). EP300-related Rubinstein-Taybi syndrome: Highlighted rare phenotypic findings and a genotype-phenotype meta-analysis of 74 patients. Am. J. Med. Genet. Part A 182 (12), 2926–2938. 10.1002/ajmg.a.61883 33043588

[B17] Coupry I. Roudaut C. Stef M. Delrue M. Marche M. Burgelin I. (2002). Molecular analysis of the CBP gene in 60 patients with Rubinstein-Taybi syndrome. J. Med. Genet. 39 (6), 415–421. 10.1136/jmg.39.6.415 12070251 PMC1735143

[B19] Crawford H. Waite J. Oliver C. (2017). Diverse profiles of anxiety related disorders in fragile X, cornelia de Lange and rubinstein-taybi syndromes. J. Autism Dev. Disord. 47 (12), 3728–3740. 10.1007/s10803-016-3015-y 28144878 PMC5676833

[B20] Douzgou S. Dell'Oro J. Fonseca C. R. Rei A. Mullins J. Jusiewicz I. (2022). The natural history of adults with rubinstein-taybi syndrome: A families-reported experience. Eur. J. Hum. Genet. 30 (7), 841–847. 10.1038/s41431-022-01097-8 35388185 PMC9259744

[B21] Ellis K. Oliver C. Stefanidou C. Apperly I. Moss J. (2020). An observational study of social interaction skills and behaviors in cornelia de Lange, fragile X and rubinstein-taybi syndromes. J. Autism Dev. Disord. 50 (11), 4001–4010. 10.1007/s10803-020-04440-4 32189229 PMC7560922

[B22] Equit M. Piro-Hussong A. Niemczyk J. Curfs L. von Gontard A. (2013). Elimination disorders in persons with Prader-Willi and Fragile-X syndromes. Neurourol. urodynamics 32 (7), 986–992. 10.1002/nau.22352 23239431

[B23] Fergelot P. Van Belzen M. Van Gils J. Afenjar A. Armour C. Arveiler B. (2016). Phenotype and genotype in 52 patients with Rubinstein-Taybi syndrome caused by EP300 mutations. Am. J. Med. Genet. Part A 170 (12), 3069–3082. 10.1002/ajmg.a.37940 27648933

[B24] Fisch G. S. Simensen R. J. Schroer R. J. (2002). Longitudinal changes in cognitive and adaptive behavior scores in children and adolescents with the fragile X mutation or autism. J. Autism Dev. Disord. 32 (2), 107–114. 10.1023/a:1014888505185 12058838

[B25] Galéra C. Taupiac E. Fraisse S. Naudion S. Toussaint E. Rooryck-Thambo C. (2009). Socio-behavioral characteristics of children with rubinstein-taybi syndrome. J. Autism Dev. Disord. 39 (9), 1252–1260. 10.1007/s10803-009-0733-4 19350377

[B26] Giani L. Michelini G. Ajmone P. F. Scaini S. Selicorni A. Vizziello P. (2022). Age-related hallmarks of psychopathology in Cornelia de Lange and Rubinstein-Taybi syndromes. Res. Dev. Disabil. 126, 104235. 10.1016/j.ridd.2022.104235 35468572

[B27] Goodman W. Grice D. Lapidus K. Coffey B. (2014). Obsessive-compulsive disorder. Psychiatric Clin. N. Am. 37 (3), 257–267. 10.1016/j.psc.2014.06.004 25150561

[B28] Goodman W. K. Price L. H. (1992). Assessment of severity and change in obsessive compulsive disorder. Psychiatr. Clin. North Am. 15 (4), 861–869. 10.1016/s0193-953x(18)30214-4 1461801

[B29] Gotts E. Liemohn W. (1977). Behavioral characteristics of three children with the broad thumb-hallux (Rubinstein-Taybi) syndrome. Biol. psychiatry 12 (3), 413–423. Available at: https://www.ncbi.nlm.nih.gov/pubmed/871491 . 871491

[B30] Graf M. D. Needham D. F. Teed N. Brown T. (2013). Genetic testing insurance coverage trends: A review of publicly available policies from the largest US payers. Pers. Med. 10 (3), 235–243. 10.2217/pme.13.9 29768740

[B31] Grant P. Langlois S. Lynd L. D. Study G. Austin J. C. Elliott A. M. (2021). Out-of-pocket and private pay in clinical genetic testing: A scoping review. Clin. Genet. 100 (5), 504–521. 10.1111/cge.14006 34080181

[B32] Hooper S. R. Curtiss K. Fau - Schoch K. Schoch K. Fau - Keshavan M. S. Keshavan Ms Fau - Allen A. (2013). A longitudinal examination of the psychoeducational, neurocognitive, and psychiatric functioning in children with 22q11.2 deletion syndrome. Res. Dev. Disabil. 34, 1758–1769. (1873-3379 (Electronic)). 10.1016/j.ridd.2012.12.003 23506790 PMC3783943

[B33] Imaizumi K. Kuroki Y. (1991). Rubinstein-Taybi syndrome with de novo reciprocal translocation t(2;16)(p13.3;p13.3). Am. J. Med. Genet. 38 (4), 636–639. 10.1002/ajmg.1320380430 2063911

[B34] Kaat A. Lecavalier L. Aman M. (2014). Validity of the aberrant behavior checklist in children with autism spectrum disorder. J. Autism Dev. Disord. 44 (5), 1103–1116. 10.1007/s10803-013-1970-0 24165702

[B35] Kessler R. C. Berglund P Fau - Demler O. Demler O Fau - Jin R. Jin R. Fau - Merikangas K. R. Merikangas Kr Fau - Walters E. E. (2005). Lifetime prevalence and age-of-onset distributions of DSM-IV disorders in the national comorbidity survey replication. Arch. Gen. Psychiatry 62, 593–602. (0003-990X (Print)). 10.1001/archpsyc.62.6.593 15939837

[B36] Lacombe D. Saura R. Taine L. Battin J. (1992). Confirmation of assignment of a locus for Rubinstein-Taybi syndrome gene to 16p13.3. Am. J. Med. Genet. 44 (1), 126–128. 10.1002/ajmg.1320440134 1519642

[B37] Levitas A. Reid C. (1998). Rubinstein-Taybi syndrome and psychiatric disorders. J. Intellect. Disabil. Res. 42 (4), 284–292. 10.1046/j.1365-2788.1998.00136.x 9786443

[B38] Masuno M. Imaizumi K. Kurosawa K. Makita Y. Petrij F. Dauwerse H. G. (1994). Submicroscopic deletion of chromosome region 16p13.3 in a Japanese patient with Rubinstein-Taybi syndrome. Am. J. Med. Genet. 53 (4), 352–354. 10.1002/ajmg.1320530409 7864045

[B39] Moss J. Nelson L. Powis L. Waite J. Richards C. Oliver C. (2016). A comparative study of sociability in angelman, cornelia de Lange, fragile X, down and rubinstein taybi syndromes and autism spectrum disorder. Am. J. Intellect. Dev. Disabil. 121 (6), 465–486. 10.1352/1944-7558-121.6.465 27802104

[B40] Nauta M. H. Scholing A. Rapee R. M. Abbott M. Spence S. H. Waters A. (2004). A parent-report measure of children's anxiety: Psychometric properties and comparison with child-report in a clinic and normal sample. Behav. Res. Ther. 42 (7), 813–839. 10.1016/S0005-7967(03)00200-6 15149901

[B41] Negri G. Milani D. Colapietro P. Forzano F. Della M. M. Rusconi D. (2015). Clinical and molecular characterization of Rubinstein-Taybi syndrome patients carrying distinct novel mutations of the EP300 gene. Clin. Genet. 87 (2), 148–154. 10.1111/cge.12348 24476420

[B42] Ogryzko V. V. Schiltz R. L. Russanova V. Howard B. H. Nakatani Y. (1996). The transcriptional coactivators p300 and CBP are histone acetyltransferases. Cell 87 (5), 953–959. 10.1016/S0092-8674(00)82001-2 8945521

[B43] Petrif F. Giles R. H. Dauwerse H. G. Saris J. J. Hennekam R. C. M. Masuno M. (1995). Rubinstein-Taybi syndrome caused by mutations in the transcriptional co-activator CBP. Nature 376 (6538), 348–351. 10.1038/376348a0 7630403

[B44] Roelfsema J. H. White S. J. Ariyürek Y. Bartholdi D. Niedrist D. Papadia F. (2005). Genetic heterogeneity in rubinstein-taybi syndrome: Mutations in both the CBP and EP300 genes cause disease. Am. J. Hum. Genet. 76 (4), 572–580. 10.1086/429130 15706485 PMC1199295

[B45] Rubinstein J. Taybi H. (1963). Broad thumbs and toes and facial abnormalities. A possible mental retardation syndrome. Am. J. Dis. Child. 105, 588–608. 10.1001/archpedi.1963.02080040590010 13983033

[B46] Sadeh A. Gruber R. Raviv A. (2002). Sleep, neurobehavioral functioning, and behavior problems in school-age children. Child. Dev. 73 (2), 405–417. 10.1111/1467-8624.00414 11949899

[B47] Shaffer R. C. Reisinger D. L. Schmitt L. M. Lamy M. Dominick K. C. Smith E. G. (2022). Systematic review: Emotion dysregulation in syndromic causes of intellectual and developmental Disabilities. J. Am. Acad. Child. Adolesc. Psychiatry 62, 518–557. 10.1016/j.jaac.2022.06.020 36007813

[B48] Sparrow S. Cicchetti D. Saulnier C. (2016). Vineland adaptive behavior scales–third edition (Vineland-3). Circle Pines, MN: American Guidance Service.

[B49] Spena S. Milani D. Rusconi D. Negri G. Colapietro P. Elcioglu N. (2015). Insights into genotype-phenotype correlations from CREBBP point mutation screening in a cohort of 46 Rubinstein-Taybi syndrome patients. Clin. Genet. 88 (5), 431–440. 10.1111/cge.12537 25388907

[B50] Spence S. H. (1999). Spence children’s anxiety scale (parent version). Brisbane: University of Queensland.

[B51] Stevens C. Carey J. Blackburn B. (1990). Rubinstein-Taybi syndrome: A natural history study. Am. J. Med. Genet. 37 (6), 30–37. 10.1002/ajmg.1320370605 2118775

[B52] Stevens C. Pouncey J. Knowles D. (2011). Adults with rubinstein-taybi syndrome. Am. J. Med. Genet. Part A 155A (7), 1680–1684. 10.1002/ajmg.a.34058 21671385

[B53] Stevens C. (2019). “Rubinstein-Taybi syndrome,” in GeneReviews. Editors Adam M. P. Mirzaa G. M. Pagon R. A. (Seattle: University of Washington). 20301699

[B54] Tommerup N. Van Der Hagen C. B. Heiberg A. (1992). Tentative assignment of a locus for Rubinstein-Taybi syndrome to 16p13.3 by a de novo reciprocal translocation, t(7;16)(q34;p13.3). Am. J. Med. Genet. 44 (2), 237–241. 10.1002/ajmg.1320440223 1456298

[B55] Valleni-Basile L. A. Garrison C. Z. Jackson K. L. Waller J. L. McKeown R. E. Addy C. L. (1994). Frequency of obsessive-compulsive disorder in a community sample of young adolescents. J. Am. Acad. Child. Adolesc. Psychiatry 33, 782–791. (0890-8567 (Print)). 10.1097/00004583-199407000-00002 8083134

[B56] Verhoeven W. Tuinier S. Kuijpers H. Egger J. Brunner H. (2010). Psychiatric profile in rubinstein-taybi syndrome. A review and case report. Psychopathology 43 (1), 63–68. 10.1159/000260045 19940543

[B57] Waite J. Moss J. Beck S. R. Richards C. Nelson L. Arron K. (2015). Repetitive behavior in rubinstein–taybi syndrome: Parallels with autism spectrum phenomenology. J. Autism Dev. Disord. 45 (5), 1238–1253. 10.1007/s10803-014-2283-7 25491025

[B58] Weissman M. M. Bland Rc Fau - Canino G. J. Canino Gj Fau - Greenwald S. Greenwald S Fau - Hwu H. G. Hwu Hg Fau - Lee C. K. Lee Ck Fau - Newman S. C. (1994). The cross national epidemiology of obsessive compulsive disorder. The Cross National Collaborative Group. J. Clin. Psychiatry 55, 5–10. 8077177

[B59] Whiteside S. P. Brown A. M. (2008). Exploring the utility of the Spence Children's Anxiety Scales parent- and child-report forms in a North American sample. J. Anxiety Disord. 22 (8), 1440–1446. 10.1016/j.janxdis.2008.02.006 18395408

[B60] Wiley S. (2020). Rubinstein-Taybi syndrome medical guidelines. Wiley - 2003 - American Journal of Medical Genetics Part A - Wiley Online Library. 10.1002/ajmg.a.10009 12749047

[B61] Wiley S. Swayne S. Rubinstein J. Lanphear N. Stevens C. (2003). Rubinstein-Taybi syndrome medical guidelines. Am. J. Med. Genet. Part A 119A (2), 101–110. 10.1002/ajmg.a.10009 12749047

[B62] Wincent J. Luthman A. van Belzen M. van der Lans C. Albert J. Nordgren A. (2016). CREBBP and EP300 mutational spectrum and clinical presentations in a cohort of Swedish patients with Rubinstein–Taybi syndrome. Mol. Genet. Genomic Med. 4 (1), 39–45. 10.1002/mgg3.177 26788536 PMC4707034

[B63] Yagihashi T. Kosaki K. Okamoto N. Mizuno S. Kurosawa K. Takahashi T. (2012). Age-dependent change in behavioral feature in Rubinstein-Taybi syndrome. Congenit. anomalies 52 (2), 82–86. 10.1111/j.1741-4520.2012.00356.x 22639993

