# Supplementary material for: Behavioral and neuropsychiatric challenges across the lifespan in individuals with Rubinstein-Taybi syndrome
Source: Front Genet. 2023 Jun 21;14:1116919. doi: 10.3389/fgene.2023.1116919 (PMC10321757; doi:10.3389/fgene.2023.1116919)
Supplement: Supplementary file 1 [file Table1.docx]

Supplementary Material

**1 Supplementary Data**

**1.1 Clinical Characteristics Definitions**

**1.12 Neuropsychological**

- Intellectual / Developmental Disability
  - Cognitive developmental delay
- Autism Spectrum Disorder (ASD)
- Attention Deficit Hyperactivity Disorder (ADHD)
- Gross motor delay
- Fine motor delay
- Speech delay
- Seizures
- Hypotonia
- Microcephaly
- Behavioral Issues
  - Self-harm
  - Aggression toward themselves
  - Increased water drinking
  - Biting
  - Head banging
  - Attention
  - Aggression toward others
  - Temper tantrums
  - Unexplained sadness
  - Increased eating with need to closely manage how much they eat
  - Scratching
  - Loss of interest in things that used to interest him/her
  - Tourette syndrome
  - Loss of interest in things that used to interest him/her
  - Loss of skills that they used to be able to perform
  - Pinching
  - Head banging
  - Banging her teeth on any surface she can find
  - Aggression
  - Rocking head side to side
  - Body rocking to self soothe
- Psychiatric Diagnosis
  - Anxiety
  - Depression
  - Obsessive Compulsive Disorder (OCD)
  - Mood disorder
  - Disruptive mood dysregulation disorder (DMDD)
  - Bipolar disorder
  - Oppositional defiant disorder (ODD)
  - Conduct disorder
- Sleep Issues
  - Difficulty staying asleep
  - Increased need for sleep
  - Sleep apnea
  - Periodic limb movement disorder
  - Sleep issues related to feeding/vomiting
  - Difficulty falling asleep
  - Insomnia
  - Aspirating during sleep causing coughing
  - Restless Legs syndrome
  - Night terrors
  - Never a good napper even from birth
  - Awful sleep for first 2.5 years while on continuous tube feeds
- Central Nervous System Malformations
  - Brain birth defect
  - Brain malformation
  - Tethered cord
  - Smaller cerebellum
  - Minimal corpus callosum
  - Hypoplastic corpus callosum
  - Agenesis of corpus callosum
  - Gray and white matter were somewhat mixed
  - Partial agenesis of the corpus callosum
  - Dysgenesis of the corpus callous
  - Spina bifida occulta
  - Mild enlarged ventricle
  - Chiari Type II malformation
  - Mild cerebellar ectopia
  - Mild flattening of the lower pons and upper medulla without soft tissue mass
  - Ventriculomegaly of 1-3

**1.2** **Scoring Methods**

**1.2.1 Y-BOCS**

Each question on the Y-BOCS is scored from 0 to 4 to yield an obsessive thoughts sub-score for questions 1-5 and a compulsive behaviors sub-score for questions 6-10. The 2 sub-scores are added together to yield a total number between 0 and 40, with 40 being the most extreme OCD-like symptoms. Based on the total score, each individual was given an approximate index of overall severity of their symptoms: 0-7 = subclinical, 8-15 = mild, 16-23 = moderate, 24-31 = severe, 32-40 = extreme.

**1.2.2 SCAS**

The 38 scenarios described on the SCAS are rated on the following Likert scale: 0= Never; 1= Sometimes; 2= Often; 3=Always. The 38 questions resolve into 6 subscales: 1. panic attack and agoraphobia (9 items - 12, 19, 25, 27, 28, 30, 32, 33, 34), 2. separation anxiety (6 items - 5, 8, 11, 14, 15, 38), 3. physical injury fears (5 items - 2, 16, 21, 23, 29), 4. social phobia (6 items - 6, 7, 9, 10, 26, 31), 5. obsessive compulsive (6 items - 13, 17, 24, 35, 36, 37), 6. generalized anxiety disorder (GAD)/overanxious disorder (6 items - 1, 3, 4, 18, 20, 22). Subscores are calculated by summing the items for each subscale.

**1.2.3 ABC-2**

The 58 behaviors described on the ABC-2 are rated on the following Likert scale: 0 = Not at all a problem; 1 = The behavior is a problem, but slight in degree; 2 = The problem is moderately serious; 3 = The problem is severe in degree. The 58 behaviors assessed are divided into five subscales: 1. irritability (15 items - 2, 4, 8, 10, 14, 19, 25, 29, 34, 36, 41, 47, 50, 52, 57), 2. social withdrawal (16 items - 3, 5, 12, 16, 20, 23, 26, 30, 32, 37, 40, 42, 43, 53, 55, 58), 3. stereotypic behavior (7 items - 6, 11, 17, 27, 35, 45, 49), 4. hyperactivity/noncompliance (16 items - 1, 7, 13, 15, 18, 21, 24, 28, 31, 38, 39, 44, 48, 51, 54, 56), and 5. inappropriate speech (4 items - 9, 22, 33, 46). Subscores are calculated by summing the items for each subscale.

**1.2.4 Vineland**

All 381 items of the Vineland are rated on the following Likert scale: 0= Never; 1= Sometimes; 2=Usually, which translates into how likely a child can complete a specific action. Each individual is given a total adaptive behavior score, a score for the 3 domains, and a score for each of the 3 subdomains within each domain, which yields a total of 13 scores. A standard score (SS) was reported for the total adaptive behavioral composite and for the 3 domain levels, which are adjusted based on typical functioning of other individuals of the same age and reported with a normative mean of 100 and a normal standard deviation of 15. A standardized v-scale score (vS) was reported for each of the subdomains, which is adjusted based on typical functioning of other individuals of the same age and based on a mean of 15 and a standard deviation of 3.

**2 Supplementary Figures and Tables**

**2.1 Supplementary Tables**

**Supplementary Table S1. Variants Reported in Participants with a Genetically Confirmed Diagnosis of RSTS.**

| ***Participant Number*** | ***Participant Age (y)*** | ***Participant Sex*** | ***Gene*** | ***Variant*** | ***Interpretation*** |
| --- | --- | --- | --- | --- | --- |
| 1 | 9.89 | M | *CREBBP* | deletion of exons 6-9 | Pathogenic |
| 2 | 13.9 | M | *CREBBP* | c.1293_1308del16insA | Pathogenic |
| 3 | 10.8 | F | *CREBBP* | c.2512delC | Pathogenic |
| 4 | 23.87 | F | *CREBBP* | c.4226C>T | Pathogenic |
| 5 | 13.29 | M | *CREBBP* | c.2464-6T>A | Pathogenic |
| 6 | 15.15 | F | *CREBBP* | c.1828C>G | Pathogenic |
| 7 | 11.87 | M | *CREBBP* | c.1225delT | Pathogenic |
| 8 | 24.04 | M | *CREBBP* | Sequencing and del/dup negative, positive DNA methylation testing for RSTS1 | -- |
| 9 | 7.41 | F | *CREBBP* | c.5051C>A (p.Ser681Tyr), positive DNA methylation testing for RSTS1 | VUS - Pathogenic |
| 10 | 9.01 | M | *CREBBP* | 16p13.3 deletion | Pathogenic |
| 11 | 3.60 | M | *CREBBP* | p.K16Ifs*2 | Pathogenic |
| 12 | 29.16 | M | *CREBBP* | c.3384dup (p.Val1129Argfs*40) | Pathogenic |
| 13 | 9.41 | F | *CREBBP* | c.3337C>T (p.Q1113X) | Pathogenic |
| 14 | 8.39 | F | *CREBBP* | c.2826delT (p.Gln943Serfs*55) | Pathogenic |
| 15 | 10.32 | M | *CREBBP* | c.904delA | Pathogenic |
| 16 | 8.03 | F | *CREBBP* | arr[hg19] 16p13.3 (3, 827, 552-3, 849,208) x3 | Pathogenic |
| 17 | 19.42 | M | *CREBBP* | c.4022G>C (p.R1341P) | Pathogenic |
| 18 | 4.15 | F | *CREBBP* | deletion of exons 22 and 23 | Pathogenic |
| 19 | 21.17 | M | *CREBBP* | c.5602C>T (p.Arg1868Trp) | Pathogenic |
| 20 | 5.75 | F | *CREBBP* | 16p13.3 deletion | Pathogenic |
| 21 | 11.20 | F | *CREBBP* | c.4492C (p.Arg1498*) | Pathogenic |
| 22 | 4.25 | M | *CREBBP* | c.886dupC | Pathogenic |
| 23 | 10.44 | M | *CREBBP* | c.86-3T>G, p.? | VUS – Likely Pathogenic |
| 24 | 15.36 | F | *CREBBP* | 16p13.3 deletion | Pathogenic |
| 25 | 11.25 | M | *CREBBP* | 16p13.3 deletion | Pathogenic |
| 26 | 17.35 | F | *CREBBP* | c.4421G>T (p.Cys1474Phe) | Pathogenic |
| 27 | 4.73 | F | *CREBBP* | 16p13.3 deletion | Pathogenic |
| 28 | 7.74 | F | *CREBBP* | c.5933A>G (p.N198S) | VUS – Likely Pathogenic |
| 29 | 10.59 | M | *CREBBP* | c.4340C>T (p.Thr1447Ile(ACA>ATA)) | Pathogenic |
| 30 | 12.05 | F | *CREBBP* | c.4439A>G (p.D1480G) | VUS – Likely Pathogenic |
| 31 | 13.38 | F | *CREBBP* | c.36del (p.ArgGlufs*31) | Pathogenic |
| 32 | 3.11 | F | *CREBBP* | c.1342_1343insG (p.Ser448Cysfs*4) | Pathogenic |
| 33 | 1.76 | M | *CREBBP* | c.2666C>G (p.S889*) | Pathogenic |
| 34 | 19.5 | F | *EP300* | c.1517delT | Pathogenic |
| 35 | 3.99 | M | *EP300* | c.832del (p.Thr278Leufs*39) | Pathogenic |
| 36 | 4.27 | M | *EP300* | c.6188_6239del (p.Ser2063Cysfs*54) | Pathogenic |
| 37 | 8.84 | F | *EP300* | c.3163C>T (p.R1055X) | Pathogenic |
| 38 | 5.97 | M | *EP300* | c.1A>T (p.Met?) | Pathogenic |
| 39 | 13.12 | M | *EP300* | c.1878+2T>C (p.?) | Pathogenic |
| 40 | 1.91 | F | *EP300* | 3' end deletion of exons 28-31 | Pathogenic |
| 41 | 5.01 | M | *EP300* | c.1109-1110 del (p.Arg370Hlsfs*40) | Pathogenic |
| 42 | 8.95 | M | *EP300* | c.729+1G>A | Pathogenic |
| 43 | 3.64 | F | *EP300* | arr[hg19] 22q13.1q13.2 (40999259_42125919) x1 | Pathogenic |
| 44 | 4.65 | F | *EP300* | c.4933C>T (p.R1645*) | Pathogenic |
| 45 | 11.35 | M | *EP300* | c.6968_6986dup19 (p.R2330PfsX55) | Pathogenic |
| 46 | 18.95 | M | *EP300* | c.2540delC (p.Pro847GlnfsTer3) | Pathogenic |
| 47 | 4.78 | F | *EP300* | deletion of exons 24 to 27 (p.7) | Pathogenic |
| 48 | 19.23 | M | *EP300* | c.280A>G (p.N94D) | Pathogenic |
| 49 | 15.15 | F | *EP300* | arr[hg19]22q13.2(41,483,647-41,619,710) x1 | Pathogenic |
| 50 | 19.10 | F | *EP300* | c.3014_3015dlGT p.Cys1005X | Pathogenic |
| 51 | 48.74 | F | *EP300* | c.7074_7055delinsCCACA (p.Gly2350HisfsTer52) | Pathogenic |
| 52 | 11.41 | M | *EP300* | c.7074_7055delinsCCACA (p.Gly2350HisfsTer52) | Pathogenic |

VUS: variant of unknown significance; M: male; F: female.

**Supplementary Table S2. Clinical Characteristics of RSTS Sample.**

| *Category* | *Characteristic* | *Overall*  *(n=56)*  *% (n)* | *RSTS1*  *(n=28)*  *% (n)* | *RSTS2*  *(n=17)*  *% (n)* | *Clinical*  *(n=11)*  *% (n)* |
| --- | --- | --- | --- | --- | --- |
| Neuropsychological | IDD | 95% (53) | 89% (25) | 100% (17) | 100% (11) |
|  | Gross Motor Delay | 93% (52) | 89% (25) | 94% (16) | 100% (11) |
|  | Fine Motor Delay | 93% (52) | 89% (25) | 94% (16) | 100% (11) |
|  | Speech Delay | 95% (53) | 89% (25) | 100% (17) | 100% (11) |
|  | Hypotonia | 61% (34) | 71% (20) | 59% (10) | 36% (4) |
|  | Psychiatric Diagnosis | 45% (25) | 43% (12) | 41% (7) | 55% (6) |
|  | Anxiety | 34% (19) | 29% (8) | 35% (6) | 45% (5) |
|  | OCD | 21% (12) | 25% (7) | 6% (1) | 36% (4) |
|  | Depression | 9% (5) | 14% (4) | 6% (1) | 0% (0) |
|  | Mood Disorder | 11% (6) | 14% (4) | 12% (2) | 0% (0) |
|  | DMDD | 4% (2) | 4% (1) | 6% (1) | 0% (0) |
|  | Bipolar Disorder | 5% (3) | 7% (2) | 6% (1) | 0% (0) |
|  | ODD | 11% (6) | 14% (4) | 12% (2) | 0% (0) |
|  | Conduct Disorder | 2% (1) | 4% (1) | 0% (0) | 0% (0) |
|  | ASD | 34% (19) | 43% (12) | 29% (5) | 18% (2) |
|  | ADHD | 39% (22) | 46% (13) | 41% (7) | 18% (2) |
|  | Behavioral Issues | 88% (49) | 79% (22) | 94% (16) | 100% (11) |
| Malformations | Microcephaly | 59% (33) | 64% (18) | 77% (13) | 18% (2) |
|  | Cardiac | 34% (19) | 39% (11) | 24% (4) | 36% (4) |
|  | Renal | 11% (6) | 11% (3 | 12% (2) | 9% (1) |
|  | Genitourinary | 30% (17) | 32% (9) | 12% (2) | 55% (6) |
|  | Gastrointestinal | 5% (3) | 7% (2) | 0% (0) | 9% (1) |
|  | Central Nervous System | 32% (18) | 36% (10) | 29% (5) | 27% (3) |
|  | Craniofacial | 71% (40) | 75% (21) | 59% (10) | 82% (9) |
|  | Musculoskeletal | 39% (22) | 36% (10) | 29% (5) | 64% (7) |
| Medical Issues over the Lifespan | Scoliosis | 29% (16) | 32% (9) | 12% (2) | 45% (5) |
|  | Obesity | 2% (1) | 4% (1) | 0% (0) | 0% (0) |
|  | Endocrinologic Issues | 34% (19) | 25% (7) | 65% (11) | 9% (1) |
|  | Keloids | 25% (14) | 21% (6) | 18% (3) | 45% (5) |
|  | Recurrent Infections | 34% (19) | 43% (12) | 29% (5) | 18% (2) |
|  | Sleep Issues | 80% (43) | 75% (21) | 77% (13) | 82% (9) |
|  | Failure to Thrive | 39% (22) | 43% (12) | 41% (7) | 27% (3) |
|  | Reflux | 52% (29) | 50% (14) | 59% (10) | 45% (5) |
|  | Constipation | 73% (41) | 7521 | 82% (14) | 55% (6) |
|  | Visual Acuity or Extraocular | 70% (39) | 61% (17) | 88% (15) | 64% (7) |
|  | Fracture History | 43% (24) | 43% (12) | 35% (6) | 55% (6) |
|  | Anesthesia Complications | 36% (20) | 46% (13) | 12% (2) | 45% (5) |
|  | Seizures | 18% (10) | 18% (5) | 24% (4) | 9% (1) |

IDD: intellectual and developmental disability, OCD: obsessive compulsive disorder, DMDD: disruptive mood dysregulation disorder, ODD: oppositional defiant disorder, ASD: autism spectrum disorder, and ADHD: attention deficit hyperactivity disorder.

**Supplementary Table S3. Summary Statistics of Neuropsychiatric and Behavioral Challenges in RSTS**

| Y-BOCS  *Total Score above Subclinical*  % (n) | Overall | Age Groups | | | | | | Sex | | | | |
| --- | --- | --- | --- | --- | --- | --- | --- | --- | --- | --- | --- | --- |
|  | n=56 | SA  (n=22) | A/EA  (n=18) | | Ad  (n=16) | | | Male  (n=28) | | | Female  (n=28) | |
|  | 82% (46) | 86% (19) | 83% (15) | | 75% (12) | | | 82% (23) | | | 82% (23) | |
|  | Types | | | | | | |  |  |  |  |  |
|  | RSTS1  (n=29) | RSTS2  (n=11) | | Clinical  (n=16) | | | |  |  |  |  |  |
|  | 90% (26) | 73% (8) | | 75% (12) | | | |  |  |  |  |  |
| SCAS  M (SD) | Subscale | | | | | *Our Sample*  *(1-61y)*  *n=71* | | *Our Sample*  *(9-18y)*  *n=26* | | | *Normative Sample*  *(9-18y)*  *n=85* | |
|  | Panic Attack & Agoraphobia | | | | | 2.75 (3.26) | | 2.92 (3.35) | | | 1.55 (2.40) | |
|  | Separation Anxiety | | | | | 3.08 (2.92) | | 3.15 (3.04) | | | 2.30 (2.40) | |
|  | Physical Injury Fears | | | | | 3.00 (2.62) | | 3.19 (2.68) | | | 2.45 (2.1) | |
|  | Social Phobia | | | | | 2.29 (3.17) | | 2.31 (3.31) | | | 4.5 (3.1) | |
|  | Obsessive Compulsive | | | | | 2.64 (3.05) | | 3.23 (2.73) | | | 2.78 (3.1) | |
|  | GAD/Overanxious Disorder | | | | | 3.16 (2.80) | | 3.35 (2.91) | | | 4.31 (2.3) | |
| SCAS  % (n)  *Above average scores compared to TD population* | Subscale | | | Overall | | Age Groups | | | | | | |
|  |  |  |  | n=56 | | SA  (n=22) | | A/EA  (n=18) | | | A  (n=16) | |
|  | Panic Attack & Agoraphobia | | | 52% (29) | | 45% (10) | | 56% (10) | | | 56% (9) | |
|  | Separation Anxiety | | | 45% (25) | | 45% (10) | | 44% (8) | | | 44% (7) | |
|  | Physical Injury Fears | | | 46% (26) | | 45% (10) | | 44% (8) | | | 50% (8) | |
|  | Social Phobia | | | 25% (14) | | 9% (2) | | 33% (6) | | | 38% (6) | |
|  | Obsessive Compulsive | | | 39% (22) | | 23% (5) | | 39% (7) | | | 44% (7) | |
|  | GAD/Overanxious Disorder | | | 32% (18) | | 27% (6) | | 33% (6) | | | 38% (6) | |
|  |  | | | | | | | | | | | |
|  | Subscale | | | Types | | | | Sex | | | | |
|  |  |  |  | RSTS1  (n=29) | | RSTS2  (n=11) | Clinical  (n=16) | Male  (n=28) | | | Female  (n=28) | |
|  | Panic Attack & Agoraphobia | | | 52% (15) | | 64% (7) | 44% (7) | 57% (16) | | | 46% (13) | |
|  | Separation Anxiety | | | 41% (12) | | 55% (6) | 44% (7) | 43% (12) | | | 46% (13) | |
|  | Physical Injury Fears | | | 45% (13) | | 55% (6) | 44% (7) | 50% (14) | | | 43% (12) | |
|  | Social Phobia | | | 17% (5) | | 55% (6) | 19% (3) | 25% (7) | | | 25% (7) | |
|  | Obsessive Compulsive | | | 48% (14) | | 27% (3) | 31% (5) | 39% (11) | | | 39% (11) | |
|  | GAD/Overanxious Disorder | | | 28% (8) | | 45% (5) | 31% (5) | 29% (8) | | | 36% (10) | |
| ABC-2  M (SD) | n = 59 | | | *Our Sample*  *(5-10y)* | | *ID Sample*  *(6-10y)* | *Our Sample*  *(11-14y)* | *ID Sample*  *(11-14y)* | *Our Sample*  *(15+ y)* | | *ID Sample*  *(15+ y)* | |
|  | Irritability | | | 11.50 (10.05) | | 8.50 (8.11) | 13.92 (10.58) | 9.36 (8.55) | 8.10 (7.07) | | 5.79 (6.74) | |
|  | Social Withdrawal | | | 8.11 (6.18) | | 5.47 (6.05) | 13.67 (12.49) | 6.90 (8.19) | 8.21 (7.63) | | 6.26 (7.36) | |
|  | Stereotypic Behavior | | | 6.07 (4.51) | | 2.71 (3.99) | 8.50 (6.83) | 2.27 (3.52) | 3.55 (3.39) | | 2.15 (3.41) | |
|  | Hyperactivity/Noncompliance | | | 19.44 (11.32) | | 12.92 (11.70) | 18.75 (10.15) | 13.12 (11.25) | 8.52 (6.70) | | 7.74 (8.30) | |
|  | Inappropriate Speech | | | 2.94 (2.53) | | 2.01 (2.6) | 3.92 (3.73) | 2.26 (2.67) | 2.86 (3.31) | | 1.64 (2.52) | |
| Vineland  % (n) | n = 56 | | | | | Percentile Rank | | | | | | |
|  |  |  |  |  |  | <1% | 1% ≤ n <5% | 5% ≤ n < 10% | | 10% ≤ n <20% | 20% ≤ n <30% | 30% ≤ n <40% |
|  | ABC | | | | | 45% (25) | 36% (20) | 11% (6) | | 2% (1) | -- | -- |
|  | Communication | | | | | 46% (26) | 34% (19) | 9% (5) | | 11% (6) | -- | -- |
|  | Daily Living Skills | | | | | 59% (33) | 30% (17) | 7% (4) | | -- | 4% (2) | -- |
|  | Socialization | | | | | 46% (26) | 21% (12) | 9% (5) | | 16% (9) | 4% (2) | 2% (1) |

M: mean, SD: standard deviation, SA: school age, A/EA: adolescence/early adulthood, A: adulthood, GAD: generalized anxiety disorder, and ABC: adaptive behavior composite. Summary statistics include only validated age ranges unless otherwise denoted.

**Supplementary Table S4. Summary of Significant Findings Based on Age Regression Models by RSTS Type.**

| *Measure* | *Variable* | *Type* | *Model* | *r^2^* | *F* | *p* |
| --- | --- | --- | --- | --- | --- | --- |
| ABC-2† | Irritability | Overall | Linear | 0.07 | 4.01 | 0.050 |
|  |  | Type 1 | Quadratic | 0.03 | 0.41 | 0.669 |
|  |  | Type 2 | Linear | 0.35 | 6.03 | 0.032 |
|  |  | Clinical | Quadratic | 0.13 | 0.96 | 0.408 |
|  | Stereotypic Behaviors | Overall | Linear | 0.08 | 4.72 | 0.034 |
|  |  | Type 1 | Linear | 0.00 | 0.00 | 0.991 |
|  |  | Type 2 | Quadratic | 0.25 | 1.63 | 0.244 |
|  |  | Clinical | Logarithmic | 0.11 | 1.64 | 0.221 |
|  | Hyperactivity | Overall | Logarithmic | 0.25 | 19.30 | <0.001 |
|  |  | Type 1 | Quadratic | 0.03 | 0.47 | 0.628 |
|  |  | Type 2 | Logarithmic | 0.59 | 15.76 | 0.002 |
|  |  | Clinical | Logarithmic | 0.26 | 4.85 | 0.045 |
| Vineland Domain Standard Scores and Subdomain Raw Scores | Adaptive Behavior Composite | Overall | Quadratic | 0.17 | 5.57 | 0.006 |
|  |  | Type 1 | Linear | 0.26 | 8.74 | 0.007 |
|  |  | Type 2 | Quadratic | 0.25 | 1.96 | 0.183 |
|  |  | Clinical | Quadratic | 0.15 | 0.97 | 0.408 |
|  | Communication  Domain | Overall | Linear | 0.10 | 5.90 | 0.019 |
|  |  | Type 1 | Quadratic | 0.06 | 0.72 | 0.495 |
|  |  | Type 2 | Quadratic | 0.21 | 1.63 | 0.236 |
|  |  | Clinical | Quadratic | 0.14 | 0.90 | 0.436 |
|  | Receptive  Subdomain | Overall | Logarithmic | 0.26 | 19.14 | <0.001 |
|  |  | Type 1 | Logarithmic | 0.18 | 5.59 | 0.026 |
|  |  | Type 2 | Logarithmic | 0.50 | 13.01 | 0.003 |
|  |  | Clinical | Logarithmic | 0.15 | 2.10 | 0.173 |
|  | Expressive  Subdomain | Overall | Logarithmic | 0.23 | 15.87 | <0.001 |
|  |  | Type 1 | Logarithmic | 0.28 | 9.60 | 0.005 |
|  |  | Type 2 | Logarithmic | 0.60 | 19.30 | <0.001 |
|  |  | Clinical | Quadratic | 0.12 | 0.07 | 0.936 |
|  | Written  Subdomain | Overall | Quadratic | 0.27 | 9.31 | <0.001 |
|  |  | Type 1 | Quadratic | 0.44 | 9.00 | 0.001 |
|  |  | Type 2 | Quadratic | 0.94 | 89.57 | <0.001 |
|  |  | Clinical | Quadratic | 0.16 | 0.89 | 0.916 |
|  | Daily Living Skills Domain | Overall | Quadratic | 0.12 | 3.56 | 0.035 |
|  |  | Type 1 | Linear | 0.34 | 12.93 | 0.001 |
|  |  | Type 2 | Quadratic | 0.28 | 2.33 | 0.139 |
|  |  | Clinical | Quadratic | 0.08 | 0.44 | 0.652 |
|  | Personal  Subdomain | Overall | Logarithmic | 0.36 | 31.44 | <0.001 |
|  |  | Type 1 | Quadratic | 0.32 | 5.54 | 0.011 |
|  |  | Type 2 | Logarithmic | 0.79 | 48.72 | <0.001 |
|  |  | Clinical | Logarithmic | 0.30 | 5.12 | 0.043 |
|  | Domestic  Subdomain | Overall | Logarithmic | 0.23 | 15.88 | <0.001 |
|  |  | Type 1 | Quadratic | 0.29 | 4.68 | 0.020 |
|  |  | Type 2 | Logarithmic | 0.65 | 22.63 | <0.001 |
|  |  | Clinical | Quadratic | 0.01 | 0.67 | 0.935 |
|  | Community  Subdomain | Overall | Logarithmic | 0.27 | 18.97 | <0.001 |
|  |  | Type 1 | Quadratic | 0.31 | 5.05 | 0.015 |
|  |  | Type 2 | Quadratic | 0.87 | 37.25 | <0.001 |
|  |  | Clinical | Logarithmic | 0.12 | 1.66 | 0.221 |
|  | Socialization  Domain | Overall | Quadratic | 0.30 | 11.22 | <0.001 |
|  |  | Type 1 | Linear | 0.36 | 13.89 | <0.001 |
|  |  | Type 2 | Quadratic | 0.08 | 0.52 | 0.610 |
|  |  | Clinical | Quadratic | 0.40 | 3.64 | 0.061 |
|  | Interpersonal Relationships  Subdomain | Overall | Logarithmic | 0.14 | 8.97 | 0.004 |
|  |  | Type 1 | Logarithmic | 0.38 | 0.99 | 0.328 |
|  |  | Type 2 | Logarithmic | 0.60 | 19.50 | <0.001 |
|  |  | Clinical | Quadratic | 0.21 | 1.42 | 0.283 |
|  | Play and Leisure  Subdomain | Overall | Logarithmic | 0.11 | 6.97 | 0.011 |
|  |  | Type 1 | Quadratic | .15 | 2.14 | 0.140 |
|  |  | Type 2 | Logarithmic | 0.50 | 13.01 | 0.003 |
|  |  | Clinical | Quadratic | 0.15 | 0.93 | 0.422 |
|  | Coping Skills  Subdomain | Overall | Logarithmic | 0.09 | 5.58 | 0.022 |
|  |  | Type 1 | Quadratic | 0.14 | 0.159 | 0.854 |
|  |  | Type 2 | Logarithmic | 0.62 | 20.78 | <0.001 |
|  |  | Clinical | Quadratic | 0.52 | 5.97 | 0.018 |

† - age restricted to 5-year-old participants and older for the ABC-2 measure given validated range beginning at 5 years.

**Supplementary Table S5. Neuropsychiatric and Behavioral Abilities and Challenges over RSTS Age Groups – Full Dataset**

| Measure |  | Overall  M(SD) | Early Childhood^a^  M(SD) | | School Age^b^  M(SD) | Adolescence/  Early Adulthood^c^  M(SD) | Adulthood^d^ M(SD) | F | p | Post-hoc |
| --- | --- | --- | --- | --- | --- | --- | --- | --- | --- | --- |
| Y-BOCS | n | 71 | | 15 | 22 | 18 | 16 |  |  |  |
|  | Obsessive Thoughts | 7.01 (5.17) | | 3.99 (4.25) | 6.95 (5.71) | 8.35 (4.68) | 8.41 (4.88) | 0.51 | 0.605 |  |
|  | Compulsive Behaviors | 7.43 (5.72) | | 3.58 (4.09) | 8.06 (6.00) | 8.80 (5.30) | 8.63 (5.98) | 0.09 | 0.914 |  |
|  | Total | 14.78 (10.07) | | 7.81 (7.82) | 15.68 (10.20) | 17.46 (9.03) | 17.06 (10.70) | 0.18 | 0.840 |  |
| SCAS | n | 71 | | 15 | 22 | 18 | 16 |  |  |  |
|  | Panic Attack & Agoraphobia | 2.75 (3.26) | | 2.20 (2.88) | 2.86 (3.60) | 2.33 (2.45) | 3.56 (3.97) | 0.56 | 0.576 |  |
|  | Separation Anxiety | 3.08 (2.92) | | 2.73 (2.52) | 3.50 (3.25) | 2.72 (2.20) | 3.25 (3.61) | 0.325 | 0.724 |  |
|  | Physical Injury Fears | 3.00 (2.62) | | 2.27 (2.15) | 2.95 (2.77) | 3.22 (2.73) | 3.50 (2.76) | 0.18 | 0.833 |  |
|  | Social Phobia | 2.29 (3.17) | | 1.57 (1.79) | 1.64 (3.16) | 3.17 (3.20) | 2.81 (3.94) | 1.11 | 0.336 |  |
|  | Obsessive Compulsive | 2.64 (3.05) | | 0.79 (1.19) | 2.95 (3.40) | 2.89 (2.37) | 3.56 (3.83) | 0.22 | 0.801 |  |
|  | GAD/Overanxious Disorder | 3.16 (2.80) | | 1.50 (2.18) | 3.05 (2.90) | 3.78 (2.53) | 4.06 (3.00) | 0.67 | 0.514 |  |
| ABC-2 | n | 71 | | 15 | 22 | 18 | 16 |  |  |  |
|  | Irritability | 9.76 (8.47) | | 6.87 (4.63) | 14.27 (11.02) | 7.61 (5.79) | 8.69 (7.84) | 2.53 | 0.067 |  |
|  | Social Withdrawal | 8.87 (8.25) | | 7.13 (5.68) | 11.91 (10.14) | 7.83 (7.41) | 7.50 (7.85) | 1.10 | 0.356 |  |
|  | Stereotypic Behavior | 5.34 (4.76) | | 4.93 (3.69) | 7.96 (6.09) | 3.73 (3.71) | 3.95 (3.19) | 3.71 | 0.017 | b>c**,d* |
|  | Hyperactivity/  Noncompliance | 13.52 (9.85) | | 12.13 (7.75) | 21.68 (10.48) | 9.39 (5.71) | 8.25 (7.52) | 10.06 | <0.001 | b>c***,d*** |
|  | Inappropriate Speech | 2.70 (3.09) | | 1.13 (1.92) | 3.32 (3.17) | 3.22 (3.35) | 2.75 (3.32) | 0.12 | 0.947 |  |
| Vineland Standard and Subdomain  V-scale Scores | n | 56 | | 14 | 18 | 14 | 10 |  |  |  |
|  | ABC | 58.27 (16.16) | | 64.36 (7.87) | 59.67 (17.77) | 61.21 (16.04) | 43.10 (14.39) | 4.53 | 0.007 | d < a**, b**, c** |
|  | Communication | 56.54 (19.85) | | 60.57 (14.33) | 56.44 (22.59) | 64.21 (17.08) | 40.30 (17.77) | 3.56 | 0.020 | d < a*, b*, c** |
|  | Receptive | 7.66 (3.67) | | 7.79 (3.12) | 7.17 (4.66) | 8.57 (2.95) | 7.10 (3.54) | 0.470 | 0.708 |  |
|  | Expressive | 8.21 (4.25) | | 6.57 (3.84) | 7.94 (4.93) | 10.00 (3.88) | 8.50 (3.50) | 1.61 | 0.198 |  |
|  | Written | 6.83 (3.70) | | 8.58 (3.34) | 6.50 (3.24) | 8.00 (3.44) | 3.70 (3.56) | 4.57 | 0.007 | d < a**, b*, c** |
|  | Daily Living Skills | 53.95 (17.63) | | 58.29 (14.14) | 55.94 (17.10) | 57.36 (19.57) | 39.50 (14.75) | 3.09 | 0.035 | d < a**, b*, c* |
|  | Personal | 6.34 (3.66) | | 5.79 (2.89) | 6.17 (3.88) | 7.50 (4.45) | 5.80 (3.12) | 0.650 | 0.587 |  |
|  | Domestic | 7.17 (3.37) | | 8.42 (2.68) | 7.67 (3.09) | 7.50 (3.41) | 4.30 (3.37) | 3.61 | 0.020 | d < a**, b**, c* |
|  | Community | 6.35 (3.40) | | 7.83 (2.37) | 6.39 (3.50) | 6.86 (3.70) | 3.80 (2.78) | 3.06 | 0.036 | d < a**, b*, c* |
|  | Socialization | 59.14 (19.96) | | 69.50 (10.70) | 63.11 (21.93) | 58.86 (18.65) | 37.90 (12.55) | 7.00 | <0.001 | d < a***, b***, c** |
|  | IP Relationships | 7.85 (3.62) | | 8.43 (1.87) | 8.33 (4.30) | 8.00 (4.21) | 6.00 (3.13) | 1.11 | 0.353 |  |
|  | Play and Leisure | 7.88 (3.46) | | 9.00 (2.75) | 8.11 (4.16) | 8.07 (3.43) | 5.60 (2.22) | 2.09 | 0.113 |  |
|  | Coping Skills | 8.11 (3.20) | | 10.31 (1.89) | 8.11 (3.77) | 7.29 (3.05) | 6.40 (2.27) | 3.82 | 0.015 | a > b*, c*, d** |
| Vineland Subdomain Raw Scores | n | 56 | | 14 | 18 | 14 | 10 |  |  |  |
|  | Receptive | 52.14 (17.97) | | 39.93 (19.63) | 49.06 (19.85) | 62.29 (8.20) | 60.60 (9.12) | 5.74 | 0.002 | a<c***,d**; c>b* |
|  | Expressive | 59.05 (29.96) | | 34.93 (28.05) | 59.39 (29.82) | 76.00 (21.99) | 68.50 (22.80) | 6.23 | 0.001 | a<b*,c***,d** |
|  | Written | 27.46 (19.81) | | 7.50 (7.49) | 26.06 (17.10) | 44.79 (16.79) | 29.70 (16.59) | 12.88 | <0.001 | a<b**,c***,d**; b<c**; c>d*; d>a** |
|  | Personal | 54.54 (30.16) | | 25.93 (18.21) | 52.33 (27.59) | 73.50 (26.56) | 72.00 (21.05) | 11.19 | <0.001 | a<b**,c***,d***; b<c*,d* |
|  | Domestic | 13.35 (12.64) | | 2.42 (1.98) | 11.06 (10.85) | 23.00 (12.87) | 17.10 (12.01) | 9.01 | <0.001 | a<b*,c***,d**; b<c** |
|  | Community | 27.30 (23.46) | | 5.67 (4.46) | 22.33 (18.43) | 46.43 (25.16) | 35.40 (18.61) | 11.38 | <0.001 | a<b*,c***,d***; b<c*** |
|  | IP Relationships | 42.88 (19.10) | | 29.71 (11.67) | 44.11 (22.50) | 51.64 (18.67) | 46.80 (12.78) | 3.90 | 0.014 | a<b*,c**,d* |
|  | Play and Leisure | 29.95 (15.49) | | 19.50 (9.34) | 31.06 (17.61) | 38.29 (16.29) | 30.90 (9.45) | 4.12 | 0.011 | a<b*,c** |
|  | Coping Skills | 27.29 (12.44) | | 22.00 (6.42) | 26.28 (15.12) | 31.07 (13.21) | 30.70 (10.61) | 1.55 | 0.213 |  |
| Vineland Subdomain Growth Scale Values | n | 56 | | 14 | 18 | 14 | 10 |  |  |  |
|  | Receptive | 94.59 (18.25) | | 81.64 (21.32) | 92.28 (18.70) | 104.57 (9.07) | 102.90 (9.76) | 5.70 | 0.002 | a<c***, d**, b<c* |
|  | Expressive | 82.89 (21.52) | | 65.50 (19.51) | 83.61 (21.45) | 95.36 (17.16) | 88.50 (15.41) | 6.23 | 0.001 | a<b*,c***, d** |
|  | Written | 60.11 (23.63) | | 33.75 (17.77) | 60.11 (19.12) | 79.00 (17.52) | 65.30 (16.06) | 14.17 | <0.001 | a<b***,c***, d*** |
|  | Personal | 82.95 (20.62) | | 64.29 (14.32) | 81.39 (17.00) | 96.21 (21.63) | 93.30 (11.87) | 9.82 | <0.001 | a<b**, c***,d*** |
|  | Domestic | 43.31 (18.95) | | 27.08 (8.67) | 40.22 (19.27) | 55.21 (18.56) | 51.70 (12.51) | 7.70 | <0.001 | a<b*,c***,d*** |
|  | Community | 52.76 (20.14) | | 33.67 (11.40) | 48.67 (20.42) | 67.79 (15.57) | 62.00 (11.24) | 11.31 | <0.001 | a<b*,c***,d*** |
|  | IP Relationships | 75.52 (14.57) | | 65.71 (9.36) | 76.28 (17.12) | 82.43 (14.47) | 78.20 (9.07) | 3.79 | 0.016 | a<b*,c**,d* |
|  | Play and Leisure | 64.32 (13.53) | | 56.07 (10.54) | 64.78 (15.45) | 70.86 (13.99) | 65.90 (6.98) | 3.23 | 0.030 | a<c** |
|  | Coping Skills | 62.80 (11.62) | | 58.62 (5.91) | 61.22 (14.68) | 66.29 (11.96) | 66.20 (9.39) | 1.41 | 0.251 |  |

F and P-values represent comparisons between age groups and exclude the early childhood group for the Y-BOCS and SCAS measures as well as exclude all participants under the age of 5 years for the ABC-2 measure. Post-hoc analyses represent exclusion of early childhood group for the Y-BOCS and SCAS measures as well as exclude all participants under the age of 5 years for the ABC-2 measure. All data shown for completeness in supplemental table. Asterisks represent statistical significance (* p<0.05; ** p<0.01; *** p<0. 001). M: mean, SD: standard deviation, GAD: generalized anxiety disorder, ABC: adaptive behavior composite, and IP: interpersonal.

**Supplementary Table S6. Neuropsychiatric and Behavioral Abilities and Challenges by Biological Sex**

| Measure |  | Female  M(SD) | Male  M(SD) | F | p |
| --- | --- | --- | --- | --- | --- |
| Y-BOCS | n | 28 | 28 |  |  |
|  | Obsessive Thoughts | 7.44 (5.49) | 8.20 (4.79) | 0.30 | 0.585 |
|  | Compulsive Behaviors | 8.63 (5.71) | 8.29 (5.75) | 0.05 | 0.824 |
|  | Total | 16.61 (10.20) | 16.69 (9.65) | 0.00 | 0.976 |
| SCAS | n | 28 | 28 |  |  |
|  | Panic Attack & Agoraphobia | 2.75 (3.51) | 3.04 (3.27) | 0.10 | 0.754 |
|  | Separation Anxiety | 3.39 (3.33) | 2.96 (2.74) | 0.28 | 0.601 |
|  | Physical Injury Fears | 3.04 (2.50) | 3.36 (2.95) | 0.19 | 0.662 |
|  | Social Phobia | 2.75 (3.42) | 2.18(3.45) | 0.39 | 0.536 |
|  | Obsessive Compulsive | 3.07 (2.96) | 3.14 (3.48) | 0.01 | 0.934 |
|  | GAD/Overanxious Disorder | 3.75 (2.88) | 3.39 (2.77) | 0.22 | 0.638 |
| ABC-2 | n | 29 | 30 |  |  |
|  | Irritability | 11.79 (11.01) | 8.90 (6.29) | 1.55 | 0.219 |
|  | Social Withdrawal | 9.59 (10.16) | 9.00 (6.92) | 0.07 | 0.796 |
|  | Stereotypic Behavior | 5.21 (5.73) | 5.51 (4.13) | 0.05 | 0.822 |
|  | Hyperactivity/Noncompliance | 14.31 (11.72) | 13.57 (9.08) | 0.08 | 0.786 |
|  | Inappropriate Speech | 3.17 (3.51) | 3.03 (2.85) | 0.03 | 0.868 |
| Vineland Standard and  Subdomain V-scale Scores | n | 28 | 28 |  |  |
|  | ABC | 60.75 (15.75) | 55.79 (16.46) | 1.33 | 0.254 |
|  | Communication | 59.75 (17.95) | 53.32 (21.42) | 1.48 | 0.229 |
|  | Receptive | 7.96 (3.60) | 7.36 (3.78) | 0.38 | 0.541 |
|  | Expressive | 8.07 (4.45) | 8.36 (4.13) | 0.06 | 0.804 |
|  | Written | 7.41 (3.12) | 6.26 (4.18) | 1.31 | 0.258 |
|  | Daily Living Skills | 56.29 (17.39) | 51.61 (17.87) | 0.99 | 0.325 |
|  | Personal | 6.54 (4.00) | 6.14 (3.35) | 0.16 | 0.692 |
|  | Domestic | 7.78 (3.26) | 6.56 (3.42) | 1.80 | 0.185 |
|  | Community | 6.81 (3.16) | 5.89 (3.62) | 1.00 | 0.321 |
|  | Socialization | 62.14 (20.29) | 56.14 (19.52) | 1.27 | 0.264 |
|  | IP Relationships | 8.04 (3.98) | 7.68 (3.29) | 0.13 | 0.716 |
|  | Play and Leisure | 8.25 (3.80) | 7.50 (3.12) | 0.65 | 0.423 |
|  | Coping Skills | 8.39 (3.33) | 7.81 (3.09) | 0.44 | 0.508 |
| Vineland Subdomain Raw Scores | Receptive | 51.64 (17.58) | 52.64 (18.65) | 0.43 | 0.837 |
|  | Expressive | 56.75 (31.23) | 61.36 (29.03) | 0.33 | 0.570 |
|  | Written | 25.89 (19.21) | 29.04 (20.64) | 0.34 | 0.564 |
|  | Personal | 51.50 (30.61) | 57.57 (29.95) | 0.56 | 0.456 |
|  | Domestic | 12.69 (13.38) | 13.74 (12.10) | 0.05 | 0.824 |
|  | Community | 24.30 (21.52) | 30.30 (25.30) | 0.89 | 0.352 |
|  | IP Relationships | 42.04 (20.70) | 43.71 (17.69) | 0.11 | 0.746 |
|  | Play and Leisure | 29.64 (16.42) | 30.25 (14.79) | 0.02 | 0.885 |
|  | Coping Skills | 25.64 (12.77) | 29.00 (12.09) | 1.00 | 0.322 |
| Vineland Subdomain Growth Scale Values | Receptive | 93.71 (18.73) | 95.46 (18.05) | 0.13 | 0.723 |
|  | Expressive | 81.57 (22.95) | 84.21 (20.33) | 0.21 | 0.650 |
|  | Written | 58.70 (22.77) | 61.52 (24.82) | 0.19 | 0.666 |
|  | Personal | 81.11 (22.77) | 84.79 (18.45) | 0.44 | 0.509 |
|  | Domestic | 42.07 (19.91) | 44.56 (18.24) | 0.23 | 0.635 |
|  | Community | 51.15 (18.85) | 54.37 (21.59) | 0.34 | 0.562 |
|  | IP Relationships | 74.89 (16.31) | 76.14 (12.88) | 0.10 | 0.751 |
|  | Play and Leisure | 63.14 (15.47) | 65.50 (11.43) | 0.42 | 0.519 |
|  | Coping Skills | 61.32 (12.29) | 64.33 (10.90) | 0.92 | 0.341 |

Analyses and data exclude the early childhood group for the Y-BOCS and SCAS measures as well as exclude all participants under the age of 5 years for the ABC-2 measure. M: mean, SD: standard deviation, GAD: generalized anxiety disorder, ABC: adaptive behavior composite, and IP: interpersonal.

**Supplementary Table S7. Neuropsychiatric and Behavioral Abilities and Challenges by RSTS Type**

| Measure |  | RSTS1^a^  M(SD) | RSTS2^b^  M(SD) | Clinical^c^  M(SD) | F | p | Post-hoc |
| --- | --- | --- | --- | --- | --- | --- | --- |
| Y-BOCS | n | 29 | 11 | 16 |  |  |  |
|  | Obsessive Thoughts | 8.02 (5.67) | 8.55 (3.86) | 6.96 (4.98) | 0.35 | 0.706 |  |
|  | Compulsive Behaviors | 9.84 (6.08) | 6.73 (5.35) | 7.15 (4.75) | 1.85 | 0.168 |  |
|  | Total | 18.56 (10.33) | 15.27 (8.82) | 14.13 (9.41) | 1.19 | 0.313 |  |
| SCAS | n | 29 | 11 | 16 |  |  |  |
|  | Panic Attack & Agoraphobia | 3.28 (4.02) | 3.09 (2.81) | 2.06 (2.26) | 0.69 | 0.508 |  |
|  | Separation Anxiety | 3.34 (3.38) | 3.27 (2.69) | 2.81 (2.69) | 0.16 | 0.852 |  |
|  | Physical Injury Fears | 2.97 (2.46) | 4.36 (3.80) | 2.81 (2.20) | 1.30 | 0.282 |  |
|  | Social Phobia | 2.10 (3.23) | 4.82 (4.14) | 1.50 (2.56) | 3.75 | 0.030 | a<b*; b>c* |
|  | Obsessive Compulsive | 3.62 (3.64) | 2.64 (2.50) | 2.50 (2.76) | 0.77 | 0.466 |  |
|  | GAD/Overanxious Disorder | 3.34 (3.04) | 4.64 (2.54) | 3.25 (2.49) | 0.99 | 0.377 |  |
| ABC-2 | n | 30 | 13 | 16 |  |  |  |
|  | Irritability | 12.67 (10.55) | 9.46 (6.58) | 6.63 (5.92) | 2.58 | 0.085 |  |
|  | Social Withdrawal | 9.83 (9.01) | 10.77 (8.73) | 7.06 (7.73) | 0.78 | 0.462 |  |
|  | Stereotypic Behavior | 6.91 (5.35) | 3.85 (4.62) | 3.69 (3.48) | 3.24 | 0.046 | c<a* |
|  | Hyperactivity | 17.80 (11.04) | 13.54 (8.67) | 7.00 (6.24) | 6.80 | 0.002 | a>c*** |
|  | Inapp. Speech | 3.93 (3.56) | 1.77 (2.24) | 2.63 (2.63) | 2.50 | 0.091 |  |
| Vineland Standard and Subdomain V-scale Scores | n | 27 | 15 | 14 |  |  |  |
|  | ABC | 57.19 (16.73) | 68.80 (9.64) | 49.07 (14.93) | 6.65 | 0.003 | b>a*,c*** |
|  | Communication | 53.00 (19.00) | 71.27 (12.56) | 47.57 (20.31) | 7.37 | 0.001 | b>a**,c*** |
|  | Receptive | 6.70 (3.61) | 9.67 (3.04) | 7.66 (3.67) | 3.50 | 0.037 | b>a* |
|  | Expressive | 6.67 (4.27) | 10.53 (3.62) | 8.71 (3.81) | 4.66 | 0.014 | b>a** |
|  | Written | 6.19 (3.14) | 9.71 (3.07) | 5.14 (3.82) | 7.62 | 0.001 | b>a**, c*** |
|  | Daily Living Skills | 52.96 (15.52) | 64.87 (17.12) | 44.14 (16.57) | 6.01 | 0.004 | b>a*, c** |
|  | Personal | 5.30 (3.21) | 8.53 (4.27) | 6.00 (2.91) | 4.32 | 0.018 | b>a** |
|  | Domestic | 7.08 (2.86) | 8.86 (3.16) | 5.64 (3.88) | 3.51 | 0.037 | b>c* |
|  | Community | 6.00 (2.76) | 9.21 (3.09) | 4.14 (2.93) | 11.15 | <0.001 | b>a**, c*** |
|  | Socialization | 60.81 (21.82) | 69.87 (12.93) | 44.43 (13.47) | 7.50 | 0.001 | b>c*** |
|  | IP Relationships | 7.70 (3.84) | 9.47 (3.34) | 6.43 (2.95) | 2.76 | 0.072 |  |
|  | Play and Leisure | 7.96 (3.90) | 9.20 (2.88) | 6.29 (2.58) | 2.74 | 0.074 |  |
|  | Coping Skills | 7.73 (3.74) | 10.07 (2.34) | 6.71 (1.73) | 4.95 | 0.011 | b>a*,c** |
| Vineland Subdomain Raw Scores | n | 27 | 15 | 14 |  |  |  |
|  | Receptive | 46.37 (17.96) | 55.67 (19.89) | 59.50 (12.35) | 3.07 | 0.055 | a<c* |
|  | Expressive | 48.11 (29.63) | 69.73 (30.33) | 68.71 (23.90) | 3.84 | 0.028 | a<b*,c* |
|  | Written | 20.15 (16.99) | 34.86 (22.87) | 33.64 (17.74) | 3.79 | 0.029 | a<b*,c* |
|  | Personal | 42.56 (25.97) | 62.73 (33.99) | 68.86 (25.67) | 4.86 | 0.012 | a<b*,c** |
|  | Domestic | 8.58 (9.35) | 16.29 (14.68) | 19.29 (13.25) | 4.23 | 0.020 | a<c** |
|  | Community | 17.65 (15.81) | 39.00 (31.78) | 33.50 (19.47) | 5.12 | 0.009 | a<b**,c* |
|  | IP Relationships | 39.22 (18.61) | 46.47 (24.23) | 46.07 (12.82) | 0.95 | 0.392 |  |
|  | Play and Leisure | 27.44 (15.48) | 32.87 (17.94) | 31.64 (12.76) | 0.70 | 0.503 |  |
|  | Coping Skills |  | 31.53 (13.42) | 30.00 (8.61) | 2.64 | 0.081 | a<b* |
| Vineland Subdomain Growth Scale Values | n | 27 | 15 | 14 |  |  |  |
|  | Receptive | 89.07 (16.78) | 97.40 (22.82) | 102.21 (12.23) | 2.81 | 0.069 |  |
|  | Expressive | 75.19 (19.61) | 90.53 (24.97) | 89.57 (16.72) | 3.68 | 0.032 | b>a*; c>a* |
|  | Written | 51.38 (22.71) | 67.36 (26.49) | 69.07 (16.97) | 3.80 | 0.029 | b>a*; c>a* |
|  | Personal | 75.52 (16.01) | 88.93 (27.61) | 90.86 (15.53) | 3.76 | 0.030 | b>a*; c>a* |
|  | Domestic | 36.00 (18.10) | 47.29 (19.54) | 52.93 (15.14) | 4.59 | 0.015 | c>a* |
|  | Community | 45.77 (17.22) | 59.86 (24.33) | 58.64 (17.38) | 3.30 | 0.045 | b>a* |
|  | IP Relationships | 72.78 (13.97) | 78.40 (19.19) | 77.71 (9.03) | 0.93 | 0.402 |  |
|  | Play and Leisure | 62.48 (13.20) | 66.40 (16.31) | 65.64 (11.20) | 0.48 | 0.619 |  |
|  | Coping Skills | 59.00 (12.36) | 66.73 (11.98) | 65.64 (7.58) | 2.85 | 0.067 |  |

Analyses and data exclude the early childhood group for the Y-BOCS and SCAS measures. Analyses include only 5-year-old children from the early childhood group for the ABC-2 measure. P-values represent comparisons between individuals with RSTS1 and RSTS2. Asterisks represent statistical significance (* p<0.05; ** p<0.01; *** p<0.001). M: mean, SD: standard deviation, GAD: generalized anxiety disorder, ABC: adaptive behavior composite, and IP: interpersonal.

**Supplementary Table S8. Behavioral and Psychological Drugs Reported in RSTS Sample.**

| *Brand Name* | *Generic Name* | *Indication(s)* |
| --- | --- | --- |
| Seroquel | Quetiapine | Mental/mood conditions (e.g., schizophrenia, bipolar disorder) |
| Tenex | Guanfacine | ADHD |
| Prozac | Fluoxetine | Depression, panic attacks, OCD, bulimia |
| Desyrel | Trazodone | Depression |
| Dextrostat | Dextroamphetamine | ADHD |
| Lamictal | Lamotrigine | Extreme mood swings of biopolar disorder |
| Strattera | Atomoxetine | ADHD |
| Abilify | Aripiparzole | Agitation associated with schizophrenia and bipolar disorder |
| Valdoxan | Agomelatine | Major depressive disorder, generalized anxiety disorder, bipolar depression, sleep disturbances, and seasonal affective disorder |
| Buspar | Buspirone | Generalized anxiety disorder |
| Zoloft | Sertraline | Depression, panic attacks, OCD, PTSD, and social anxiety disorder |
| Ativan | Lorazepam | Anxiety |
| Perseris | Risperidone | Schizophrenia |
| Klonopin | Clonazepam | Panic disorder |
| Celexa | Citalopram | Depression |
| Diazepam | Valium | Anxiety |
| Paroxetine HCL | Paxil | Depression, panic attacks, OCD, anxiety disorders, and PTSD |
| Desvenlafaxine succinate | Pristiq | Depression |
| Alprazolam | Xanax | Anxiety and panic disorders |
| Methylphenidate HCL | Concerta, Methylin | ADHD |
